# Multi-Organ Transcriptome Response of Lumpfish (*Cyclopterus lumpus*) to *Aeromonas salmonicida* Subspecies *salmonicida* Systemic Infection

**DOI:** 10.3390/microorganisms10112113

**Published:** 2022-10-26

**Authors:** Setu Chakraborty, Ahmed Hossain, Trung Cao, Hajarooba Gnanagobal, Cristopher Segovia, Stephen Hill, Jennifer Monk, Jillian Porter, Danny Boyce, Jennifer R. Hall, Gabriela Bindea, Surendra Kumar, Javier Santander

**Affiliations:** 1 Marine Microbial Pathogenesis and Vaccinology Laboratory, Department of Ocean Sciences, Memorial University of Newfoundland, St. John’s, NL A1C 5S7, Canada; 2 Cold-Ocean Deep-Sea Research Facility, Department of Ocean Sciences, Memorial University of Newfoundland, St. John’s, NL A1C 5S7, Canada; 3 Dr. Joe Brown Aquatic Research Building, Department of Ocean Sciences, Memorial University of Newfoundland, St. John’s, NL A1C 5S7, Canada; 4 Aquatic Research Cluster, CREAIT Network, Department of Ocean Sciences, Memorial University of Newfoundland, St. John’s, NL A1C 5S7, Canada; 5 INSERM, Laboratory of Integrative Cancer Immunology, 75006 Paris, France; 6 Equipe Labellisée Ligue Contre Le Cancer, 75013 Paris, France; 7 Centre de Recherche des Cordeliers, Sorbonne Université, Université de Paris, 75006 Paris, France; 8 Ocean Frontier Institute, Department of Ocean Sciences, Memorial University of Newfoundland, St. John’s, NL A1C 5S7, Canada

**Keywords:** *Aeromonas salmonicida* infection, lumpfish immunity, multiorgan transcriptomics, gene discovery

## Abstract

Lumpfish is utilized as a cleaner fish to biocontrol sealice infestations in Atlantic salmon farms. *Aeromonas salmonicida*, a Gram-negative facultative intracellular pathogen, is the causative agent of furunculosis in several fish species, including lumpfish. In this study, lumpfish were intraperitoneally injected with different doses of *A. salmonicida* to calculate the LD_50_. Samples of blood, head-kidney, spleen, and liver were collected at different time points to determine the infection kinetics. We determined that *A. salmonicida* LD_50_ is 10^2^ CFU per dose. We found that the lumpfish head-kidney is the primary target organ of *A. salmonicida*. Triplicate biological samples were collected from head-kidney, spleen, and liver pre-infection and at 3- and 10-days post-infection for RNA-sequencing. The reference genome-guided transcriptome assembly resulted in 6246 differentially expressed genes. The *de novo* assembly resulted in 403,204 transcripts, which added 1307 novel genes not identified by the reference genome-guided transcriptome. Differential gene expression and gene ontology enrichment analyses suggested that *A. salmonicida* induces lethal infection in lumpfish by uncontrolled and detrimental blood coagulation, complement activation, inflammation, DNA damage, suppression of the adaptive immune system, and prevention of cytoskeleton formation.

## 1. Introduction

Lumpfish (*Cyclopterus lumpus*) has been utilized as a cleaner fish species to biocontrol sea lice (e.g., *Lepeophtheirus salmonis*) infestations in Atlantic salmon (*Salmo salar*) farms in Atlantic Canada, Iceland, the US, the UK, and Norway [1,2,3,4]. Lumpfish cultivation is becoming an emergent aquaculture industry in the North Atlantic region [2,3,4,5,6], since its utilization significantly reduces or eliminates the application of toxic chemotherapeutics [3]. 

Bacterial diseases are a health concern for lumpfish delousing performance and survival rates in sea-cages [3,4]. *Aeromonas salmonicida,* a facultative intracellular pathogen, endemic worldwide in fresh and marine water, and the etiologic agent of furunculosis [7,8,9,10], is one of the most frequent pathogens of lumpfish [3,4,11,12]. *A. salmonicida* infection causes a cascade of events that usually result in fish death [13,14,15]. 

Recent studies have revealed that lumpfish have a similar innate immune system to other teleosts; for instance, macrophages display phagocytosis and respiratory burst [16], and IgM^+^ B-cells have phagocytic activity [17]. Proteomic analysis of lumpfish skin mucus has identified antimicrobial peptides and proteins involved in complement activation, inflammation, and pathogen lysis [18]. Transcriptomics analysis of primary macrophages infected with *Vibrio anguillarum* revealed the upregulation of genes encoding proteins involved in cell signaling, cytokines, and pathogen recognition components, e.g., toll-like receptors (TLRs), NOD-like receptors, interleukins, and several components of the complement system [19]. *Renibacterium salmoninarum* infection in lumpfish showed the upregulation of cytokines, pattern recognition receptors, iron regulation, and acute phase reactant-related genes. In contrast, cell-mediated adaptive immunity-related genes were down-regulated [20]. Vaccination studies in lumpfish indicate that the total levels of IgM in sera are lower than in salmon, but lumpfish produce specific antibodies upon immunization and can mount an effective adaptive immune response [21,22,23]. qPCR analyses showed that the oral immunization of lumpfish larvae resulted in the induction of canonical cytokines and chemokines-related genes [23]. However, *A. salmonicida* infection kinetics in lumpfish and lumpfish transcriptomic response to *A. salmonicida* infection have not been explored. Therefore, in this study, we characterized the kinetics of *A. salmonicida* infection in lumpfish and profiled the transcriptome response of head-kidney, spleen, and liver, at 3- and 10-days post-infection. The head kidney and the spleen are the major lymphoid organ in teleost [24], and the liver is involved in important biochemical processes in fish (e.g., metabolism) [25]. Besides, the liver is also considered as a lymphoid organ because non-parenchymal cells of the liver take part in antigen presentation and immunomodulatory functions [26,27,28], and these immune defense mechanisms of the liver are present in teleost fish [29].

We determined that *A. salmonicida* kill lumpfish in a dose-dependent manner, and the lethal dose 50 (LD_50_) was determined as 10^2^ colony-forming unit (CFU) per dose. We found that the lumpfish head-kidney is the primary target organ of *A. salmonicida*. Using reference genome-guided and *de novo* transcriptome assembly analysis, we identified tissue-specific gene expression profiles in the head-kidney, spleen, and liver. This study suggests that *A. salmonicida* induces lethal infection in lumpfish by a septic-like shock. Our RNA-Seq analysis suggests that uncontrolled and detrimental blood coagulation, complement activation, and inflammation could cause a septic-like shock which leads to hypoxia, internal organ hemorrhages, and suppression of the adaptive immune system. Our analysis also suggests an impairment of the DNA repair system, which results in cell cycle arrest and death. This relates to the type-III secretion system effectors described in *A. salmonicida*, which destabilize the cytoskeleton structure by depolymerizing actin and microtubules and inducing apoptosis [30]. Similarly, our gene ontology enrichment analysis indicates that downregulated genes in the spleen are associated with cytoskeleton structure formation, and upregulated genes are associated with the positive regulation of the apoptotic process. Overall, this study provides fundamental knowledge to understand the *A. salmonicida* infection model in a marine environment and provides valuable guidance for future pathogenicity studies.

## 2. Materials and Methods

### 2.1. Bacterial Strain, Culture Media, and Reagents

Virulent *A. salmonicida* J223 (CP048223) [31,32,33] isolated from Atlantic salmon (*Salmo salar*) in 1999 was used in this study. A single colony of *A. salmonicida* J223 [34] was grown routinely in a 16 mm diameter glass tube containing 3 mL of Trypticase Soy Broth (TSB, Difco, Franklin Lakes, NJ, USA) at 15 °C with aeration (180 rpm) for 48 h. When required, TSB was supplemented with bacto agar (1.5%; Difco) and Congo-red (0.01%; Sigma-Aldrich). Bacterial growth was monitored by spectrophotometry using a Genesys 10 U.V. spectrophotometer (Thermo, USA) and by plating to count colony-forming units (CFU) mL^−1^ [35]. 

### 2.2. Bacteria Inoculum Preparation

*A. salmonicida* J223 was initially grown in 3 mL of TSB media for 48 h. Subsequently, 300 µL of fresh culture was added to a 250 mL flask containing 30 mL of TSB media and incubated for 18 h at 15 °C with aeration (180 rpm) in an orbital shaker up to an optical density (O.D. 600 nm) of 0.7 (~1 × 10^8^ CFU mL^−1^) according to the previous description [31]. The bacterial cells were harvested by centrifugation (4200× *g* for 10 min at 4 °C), washed three times with phosphate-buffered saline (PBS; 136 mM NaCl, 2.7 mM KCl, 10.1 mM Na_2_HPO_4_, 1.5 mM KH_2_PO_4_ (pH 7.2)) [36], and finally resuspended in 300 µL of PBS. The concentrated bacterial inoculum was serially diluted in PBS (1:10) and quantified by plating onto Trypticase Soy Agar (TSA) to determine CFU mL^−1^. 

### 2.3. Ethics Statement

The experiments were performed according to the Canadian Council on Animal Care guidelines and accepted by Memorial University of Newfoundland’s Institutional Animal Care Committee (protocols #18-01-JS; #18-02-JS) [37]. 

### 2.4. Fish Holding

Juvenile specific-pathogen-free lumpfish 55.9 ± 6.4 g (mean ± SD) were obtained from the Dr. Joe Brown Aquatic Research Building (JBARB) at the Department of Ocean Sciences (DOS), Memorial University of Newfoundland (MUN), Canada [37]. All the infection assays were conducted at the national certified marine AQ3 biocontainment unit at the Cold-Ocean Deep-Sea Research Facility (CDRF), DOS, MUN. Lumpfish were kept in 500 L tanks, with flow-through (7.5 L min^−1^) of filtered and U.V. treated seawater (8–10 °C), ambient photoperiod (winter-spring), and 95–110% air saturation. The fish were fed daily using a commercial diet (Skretting—Europa 15; crude protein (55%), crude fat (15%), crude fiber (1.5%), calcium (3%), phosphorus (2%), sodium (1%), vitamin A (5000 IU Kg^−1^), vitamin D (3000 IU Kg^−1^) and vitamin E (200 IU Kg^−1^)) with a ratio of 0.5% of their body weight per day.

### 2.5. Lumpfish Infection and Lethal Dose 50 (LD50) Determination

Lumpfish were transferred from JBARB to the AQ3 biocontainment facility in 500 L tanks containing 60 fish each and acclimated for 2 weeks under optimal conditions (described above). LD_50_ of *A. salmonicida* J223 was evaluated in naïve lumpfish according to established protocols in the relevant literature [38]. Briefly, the fish were anesthetized with 40 mg of MS222 (Syndel Laboratories, Vancouver, British Colombia, Canada) per liter of seawater and intraperitoneally (ip) injected with 100 μL of 10^1^, 10^2^, 10^3^, 10^4^, and 10^5^ CFU of *A. salmonicida* J223 per dose. Five independent tanks were utilized for monitoring the mortality, and five other separate tanks were used for sampling. An additional non-infected group served as a control. Fish were visually inspected for any symptoms of the disease. The LD_50_ dose was determined for *A. salmonicida* in lumpfish according to the Reed and Muench method [39] and the Karber method [40]. Kaplan-Meier estimator and Log-rank tests were used to obtain survival fractions after the challenges and determine the differences between treatments. A one-way ANOVA was followed by Tukey’s multiple comparisons test (*p* ≤ 0.05 was considered as significant). Statistical analyses and data visualization were performed using GraphPad Prism 7.0 (La Jolla, CA, USA).

### 2.6. A. salmonicida Tissue Colonization

Five fish were netted at 0, 3, 7, 10, 14, 21, and 33 days post-infection (dpi) and immediately euthanized with an overdose of MS222 (400 mg L^−1^). The head-kidney, spleen, and liver were aseptically removed, placed in the homogenizer sterile bags (Nasco whirl-pak^®^, Thermo Scientific, Madison, USA), weighed, and suspended in PBS to a final volume of 1 mL (weight: volume). Afterward, the tissues were homogenized, the suspensions were serially diluted (1:10), and the suspensions were spread onto the TSA plate. Similarly, 1 mL of blood was collected, serially diluted, and spread onto the TSA plate. The plates were incubated at 15 °C for 4 days to determine the number of *A. salmonicida* CFU per g of tissue or 1 mL of blood, respectively. The Tukey’s multiple comparisons test followed one-way ANOVA (*p* ≤ 0.05 was considered significant). Statistical analyses and data visualization were performed using GraphPad Prism 7.0 (GraphPad Software, La Jolla, CA, USA).

### 2.7. Histopathology

Sections of lumpfish head-kidney, spleen, and liver were collected from the non-infected control and infected fish at 3 and 10 dpi. Tissues were submerged in 10% phosphate saline buffered (PBS) formalin for three consecutive days at room temperature. The fixed tissues were removed from formalin and stored in PBS at 4 °C until processing for paraffin-embedded tissue block according to established procedures [41]. Tissues were sectioned, and 5 µm sections were stained with hematoxylin and eosin (Leica Biosystems, Ontario, Canada) and visualized under a light microscope (Olympus CX21, New York, NY, USA). 

### 2.8. RNA Purification

For RNA-sequencing (RNA-Seq) and real-time quantitative polymerase chain reaction (qPCR) analyses, the tissue (~100 mg) of head-kidney, spleen, and liver were sampled from three individual lumpfish (non-infected fish), and at 3, and 10 dpi with 10^4^ CFU dose^−1^, similar to other studies [42,43,44,45,46,47]. Lumpfish tissues (*n* = 27) were preserved in 500 μL of RNAlater according to the manufacturer’s instructions (Invitrogen, Carlsbad, CA, USA) until further processing. RNA was extracted from fish tissues using the mirVana RNA isolation kit (Invitrogen, Carlsbad, CA, USA) following the manufacturer’s instructions. RNA samples were treated with 2 U mL^−1^ of TURBO DNase (TURBO DNA-free™ Kit, Invitrogen, Carlsbad, CA, USA) following the manufacturer’s instructions for the complete digestion of DNA and removal of remaining DNase and divalent cations, such as magnesium and calcium. Purified RNA samples were quantified for concentration and evaluated for purity using a spectrophotometer (Genova-nano, Jenway, Stone, Staffordshire, England), and evaluated for integrity using 1% agarose gel electrophoresis (Table S1).

### 2.9. Library Preparation and RNA-Seq

For each group, three biological replicates were analyzed for a total of 27 samples (Figure S1). Library construction and sequencing services were performed at Genome Quebec, Quebec, Canada. Briefly, high-quality RNA was evaluated using a Bioanalyzer 2100 (Agilent, Santa Clara, CA, USA), and only samples with a minimum RIN of 5 proceeded to the library construction (Figure S2, Table S1). Libraries were generated from 250 ng of total RNA using the NEBNext^®^ Multiplex Oligos for Illumina^®^ (Dual Index Primers Set 1; Adapter 1: 3’-AGATCGGAAGAGCACACGTCTGAACTCCAGTCAC-5’; Adapter 2: 3’-AGATCGGAAGAGCGTCGTGTAGGGAAAGAGTGT-5’) and sequenced in a NovaSeq 6000 Sequencer (Illumina) using a NovaSeq 6000 S4 100 bp PE flow cell. The raw data were deposited in the NCBI Sequence Read Archive (SRA) (accession number PRJNA596743).

### 2.10. Reference Transcriptome Assembly and Downstream Analysis

After RNA sequencing, paired-end raw reads were mate-paired and filtered to remove low-quality reads using the CLC Genomics Workbench v20.0 (CLCGWB; Qiagen, Hilden, Germany) with default parameters (mate-paired read information, minimum distance = 1; maximum distance = 1000). Adapter trimming was done by the CLCGWB using the trim reads tool with default parameters (quality trimming, trim using quality scores, limit: 0.05, and trim ambiguous nucleotides, the maximum number of ambiguities = 2). The quality of the reads was checked using the FastQC [48], and a multiqc [49] report was generated before and after trimming. CLCGWB then mapped trimmed reads against the lumpfish genome (NCBI accession number PRJNA625538) using the RNA-Seq analysis tool. Reads mapping and transcript counts were conducted using the following settings: mismatch cost = 2, insertion and deletion costs = 3, minimum length fraction and minimum similarity fraction = 0.8, the maximum number of hits for a read = 10, and strand-specific = both. Gene expression quantification and normalization of the mapped reads were performed by an alignment-dependent expectation-maximization (EM) algorithm [50] based on the RSEM and eXpress methods [51]. The TPM values were then computed from the counts assigned to each transcript after normalization by the trimmed mean of M-values (TMM) [52]. A global correlation analysis was performed using log_2_-transformed TPM values (x + 1) of each gene, and transcripts and correlations were estimated by the Pearson method. Abundance data were subsequently subjected to differential expression analyses using the CLCGWB and the differential expression tool based on a negative binomial general linear model (GLM) [53]. A standard selection of biologically significant differentially expressed genes (DEGs) and differentially expressed transcripts (DETs) were performed with cut-off values of log2 fold-change (FC) ≥ 1 and a false discovery rate (FDR) *p* ≤ 0.05.

### 2.11. De Novo Transcriptome Assembly, Contig Abundance, and Functional Annotation

Adaptor sequences and reads ≤50 base pairs (bp) were trimmed using the Trimmomatic v0.38 [54]. Reads were assembled into a *de novo* transcriptome using the Trinity software (v2.8.4) with default parameters [55]. Assembled contigs shorter than 200 bp were excluded from the analysis. We examined the read representation of the assembly by aligning the processed reads on *de novo* assembled transcriptome using the samtools v1.9 [56] and bowtie2 v2.3.5 [57]. We also inspected the read supports for assembled transcripts using IGV v2.7.2 [58]. Furthermore, we also examined how successfully assembled protein-coding transcripts were reconstructed to full- or near full-length using the BLAST+ and Swissprot/TrEMBL. The *de novo* transcriptome assembly quality and completeness were evaluated using BUSCO version 3 [59] against a predefined set of 4584 Actinopterygian single copy orthologs from the orthoDB v9 database.

RSEM (v1.3.1) [60] was used to quantify TPMs in the trinity package. Trinotate v3.1.1, a functional annotation pipeline, was used to generate an annotation report for the potential biological function of the assembled contigs [61]. Trinotate uses the TransDecoder v5.5 [55] to identify protein-coding regions in each assembled transcript. BLAST+ variants (blastn, blastx, and blastp) against sequence databases downloaded locally (last accessed, January 2021), including RefSeq-rna, RefSeq-protein, nt, nr and SwissProt, were used to annotate the *de novo* assembled transcriptome. The transcriptomes were further annotated for remote homologs and protein domains using HMMER v3.2.1 and Pfam v3.2.1 [62,63]. The SignalP v5.0 [64] and tmhmm v2.0c [65] software tools were used to predict signal peptides and transmembrane domains, respectively. 

A differential expression (DE) analysis was performed using DESeq2 [66] and edgeR [53]. Within each pairwise comparison, only transcripts with an FDR adjusted *p*-value ≤ 0.05 were considered significantly differentially expressed. The DE analysis was conducted at the isoform level to identify the DETs in each organ at different timepoint. 

### 2.12. Gene Filtration and Gene Ontology (GO) Enrichment Analysis

DEGs identified by reference transcriptomic assembly analysis were filtered (log_2_FC ≥ 1, *p*-value ≤ 0.05) to identify the enriched Gene Ontology terms. On the other hand, DETs identified by *de novo* reference assembly analysis and edgeR analysis were utilized for the nucleotide blast against the lumpfish genome in NCBI to extract the corresponding gene symbols. Those genes were added to the Gene Ontology term enrichment analyses. To obtain an overall view, GO enrichment analysis of all DEGs of head-kidney, spleen, and liver at 3 dpi and 10 dpi were conducted by the ClueGO App [67] (2.5.8) in Cytoscape 3.9 [68] using ClueGO source files for lumpfish (Supplementary Data 1). ClueGO source files were created using a GO OBO file downloaded on 24 March 2022. Fisher’s exact test was conducted to study the enrichment of GO terms with a *p*-value cut off ≤0.05 for 3 dpi and a *p*-value cut off ≤0.00001 for 10 dpi. A differential *p*-value cut-off and the GO term fusion strategy were employed to reduce the redundancy of the GO terms and simplify the network. To obtain a global view, GO enrichment analysis of DEGs (*n* = 600 for 10 dpi; 300 most significant DEGs (lowest FDR *p*-value) from each assembly analysis) of head-kidney, spleen, and liver at 3 dpi and 10 dpi were conducted by the ClueGO. Fisher’s exact test was conducted to study the enrichment of GO terms with a *p*-value cut off ≤0.05. The GO term fusion strategy was employed. However, to explore the pathogenesis in-depth, GO enrichment analysis associated with biological processes of upregulated and downregulated DEGs in the head-kidney 3 dpi and 10 dpi, spleen 3 dpi and 10 dpi, and liver 3 dpi and 10 dpi was conducted by setting the network specificity at medium in the ClueGO. Fisher’s exact test was conducted to study the enrichment of GO terms with a *p*-value cut off ≤0.05 (3 dpi) and 0.001 (10 dpi). The GO term fusion strategy was applied.

### 2.13. Real-Time Quantitative Polymerase Chain Reaction (qPCR) Analyses

To verify the RNA-Seq analyses, expression levels of 14 genes were measured in the same 27 RNA samples that were subjected to RNA-Seq analyses. These genes were selected based on their TPM values as they were expressed in individual samples from at least one group (i.e., uninfected control, 3 dpi or 10 dpi) in all three tissues (i.e., head-kidney, liver, and spleen). In all cases, first-strand cDNA templates were synthesized, and qPCR amplifications were performed as described previously [20,23].

The sequences, amplicon sizes, and efficiencies for all primer pairs used in the qPCR analyses are presented in Table S2. Primer pairs for *il8b*, *tlr5a*, *saa5/app*, and the endogenous control transcripts were designed, and quality control (QC) was tested previously [20]. Primers new to this study were designed following the same parameters. All primer pairs used herein were (re)-subjected to QC testing [20] using a cDNA pool generated from the spleen control group and one from the spleen 10 dpi group. The calculated efficiencies are an average of the two values.

In the experimental qPCR analyses, expression levels of the genes were normalized to expression levels of two endogenous control transcripts. The fluorescence threshold cycle (C_T_) values of all 27 samples in the study were measured (in triplicate) for each of these transcripts using cDNA of 4 ng of input total RNA, and then analyzed using geNorm [69]. Based on this analysis, *eukaryotic translation initiation factor 3 subunit D* (*etf3d*) (geNorm M = 0.31) and *elongation factor 1-alpha* (*ef1a*) (geNorm M = 0.34) were selected as the two endogenous controls.

Primer QC and endogenous control selection were followed by the experimental qPCR analyses. cDNA representing 4 ng of input RNA was used as a template in the PCR reactions. On each plate, for every sample, the selected genes and endogenous controls were tested in triplicate, and a non-template control was included. The relative quantity (RQ) of each transcript was determined using the QuantStudio Real-Time PCR Software (version 1.3) (Applied Biosystems), where Ct values were normalized with both *etf3d* and *ef1a* with amplification efficiencies incorporation. For each target of interest (TOI), the sample with the lowest normalized expression (mRNA) level was set as the calibrator sample (i.e., assigned an RQ value = 1).

To compare the TPM to the RQ values, the log_2_ normalized values were utilized. Statistical regression analyses and data visualization were performed using the GraphPad Prism 7.0 (GraphPad Software, La Jolla, CA, USA).

## 3. Results

### 3.1. LD_50_ Determination and A. salmonicida Infection Kinetics in Lumpfish

**Figure 1:**
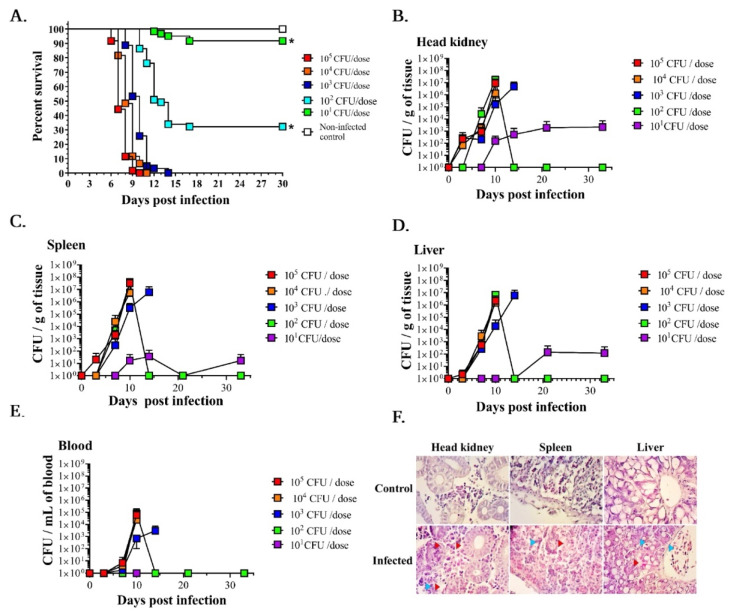
***Aeromonas salmonicida* infection in lumpfish**. (**A**). Survival of lumpfish to *A. salmonicida* infection. Lumpfish were ip injected with five different doses of *A. salmonicida* ranging from 1.1 × 10^1^ to 1.1 × 10^5^ CFU dose^−1^. The survival percentage of lumpfish infected with these different doses were 93%, 32%, 0%, 0%, and 0% at 30 dpi, respectively; (**B**–**E**). *A. salmonicida* colonization in the lumpfish lymphoid organs (head-kidney, spleen, and liver) and blood at 3, 7, 10, 21, 33 dpi; (**B**). *A. salmonicida* colonization in head-kidney; (**C**). *A. salmonicida* colonization in spleen; (**D**). *A. salmonicida* colonization in liver; (**E**). *A. salmonicida* colonization in blood; *: indicates a significant statistical difference (*p* < 0.05); (**F**). Histopathology of lumpfish tissues stained with hematoxylin and eosin. Lumpfish tissues were collected from non-infected control fish and infected fish (1.1 × 10^4^ CFU dose^−1^) at 10 dpi, visualized under the light microscope (× 400). Blue and red arrows indicate inflammatory cells and necrosis, respectively.

Five groups of 60 fish (duplicated tanks, total *n* = 600) were injected with five different doses of *A. salmonicida* J223 (10^1^, 10^2^, 10^3^, 10^4^, and 10^5^ CFU dose^−1^) to determine the LD_50_ and infection kinetics (Figure 1A). After 3 days post-infection (dpi), lack of appetite, erratic swimming, and internal hemorrhagic septicemia were observed. Fish started to die at 7–10 dpi, and there was no survival in the fish infected with the 10^3^, 10^4^, and 10^5^ CFU dose^−1^. 32% of lumpfish survived after the infection with 10^2^ CFU dose^−1^ (Figure 1A). In contrast, 93% of fish survived after the infection with the lowest dose tested (10^1^ CFU dose^−1^) (Figure 1A). The survivors and the non-infected control fish showed no symptoms of disease or mortality. The LD_50_ for *A. salmonicida* J223 in lumpfish was 187 CFU dose^−1^ according to the Reed and Muench method [39] and 273 CFU dose^−1^ according to the Karber method [40]. According to the log-rank (Mantel-Cox) test and log-rank test for trend, the survival curves and the trend were significantly different (*p* < 0.0001).

*A. salmonicida* colonization was determined at 0, 3, 7, 10, 14, 21, and 33 dpi in different tissues (Figure 1B–E). *A. salmonicida* was detected in the head-kidney at 3 dpi in fish infected with 10^3^–10^5^ CFU dose^−1^, but not when infected with 10^1^–10^2^ CFU dose^−1^. We did not detect bacteria in the liver, spleen, and blood at 3 dpi in lumpfish injected with the lowest doses (10^1^–10^4^ CFU dose^−1^) (Figure 1C–E). In contrast, a few fish infected with the 10^5^ CFU dose^−1^ showed bacterial colonization in the spleen and liver at 3 dpi (Figure 1C,D). *A. salmonicida* was first detected in blood at 7 dpi (Figure 1E). At 7 dpi, head-kidney, spleen, and liver showed bacterial colonization in all doses tested except in the lowest doses evaluated (10^1^ and 10^2^ CFU dose^−1^) (Figure 1B–D). At 10 dpi, bacterial colonization was detected in all the tissues, except for the fish infected with 10^1^ CFU dose^−1^ (Figure 1B–E). Significant differences between bacterial loads were observed only in the spleen samples of the fish infected with 10^5^ CFU dose^−1^ compared to the other groups (*p* < 0.003). At 14 dpi, 3 fish infected with the 10^3^ CFU dose^−1^ showed bacterial colonization in all tissues sampled (Figure 1B–E). After 15 dpi, no bacteria were detected in the remaining survivors in the 10^2^ CFU dose^−1^ infected group. However, *A. salmonicida* was detected in the head-kidney, spleen, and liver until 33 dpi in fish infected with 10^1^ CFU dose^−1^. These results suggest that *A. salmonicida* targets the head-kidney and disseminates to other organs, causing a systemic infection. 

Histopathological analysis indicated that *A. salmonicida* caused inflammation and tissue necrosis in head-kidney, spleen, and liver (Figure 1F). Previously, we observed intracellular *A. salmonicida* in lumpfish tissues at 3 and 10 dpi [70], and similar observations were determined in the current study.

### 3.2. Raw Sequencing Data and Quality Statistics

RNA samples were collected from the head-kidney, spleen, and liver of three non-infected lumpfish and three infected lumpfish (10^4^ CFU dose^−1^) at 3 and 10 dpi (Figure S1). RNA quality is shown in Supplementary Files (Table S1 and Figure S2A–C). RNA sequencing generated 1.08 billion Illumina NovaSeq reads ranging from 67–95 million raw reads per sample (Table S3), with a length of 101 bp. After trimming, reads were subjected to reference genome-based transcriptome assembly analysis and *de novo* transcriptome assembly analysis (Figure S1).

### 3.3. Global Profile of Differentially Expressed Genes and Transcripts Identified Using the Lumpfish Reference Genome

**Figure 2:**
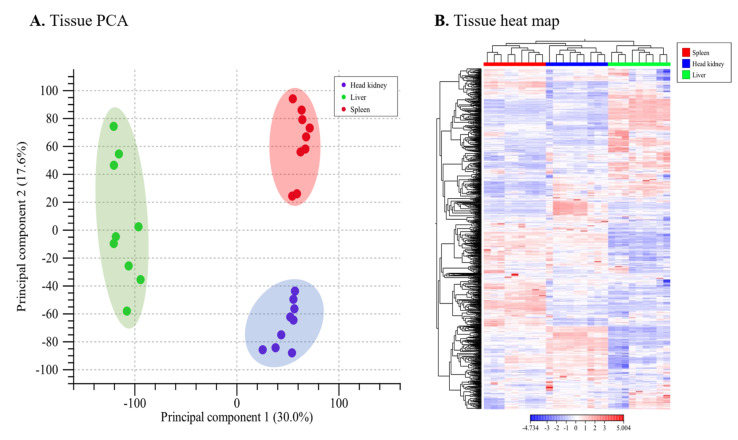
**Clusterization of gene expression profile in different lumpfish organs infected with *A. salmonicida***. (**A**). Principal component analysis (PCA) of lumpfish head-kidney, spleen, and liver infected with *A. salmonicida*; blue dot represents nine head-kidney tissue samples, red dot represents nine spleen tissue samples, green dot represents nine liver tissue samples; (**B**). Heat map of differential expressed genes of lumpfish head-kidney (blue), spleen (red), and liver (green) infected with *A. salmonicida*.

**Figure 3:**
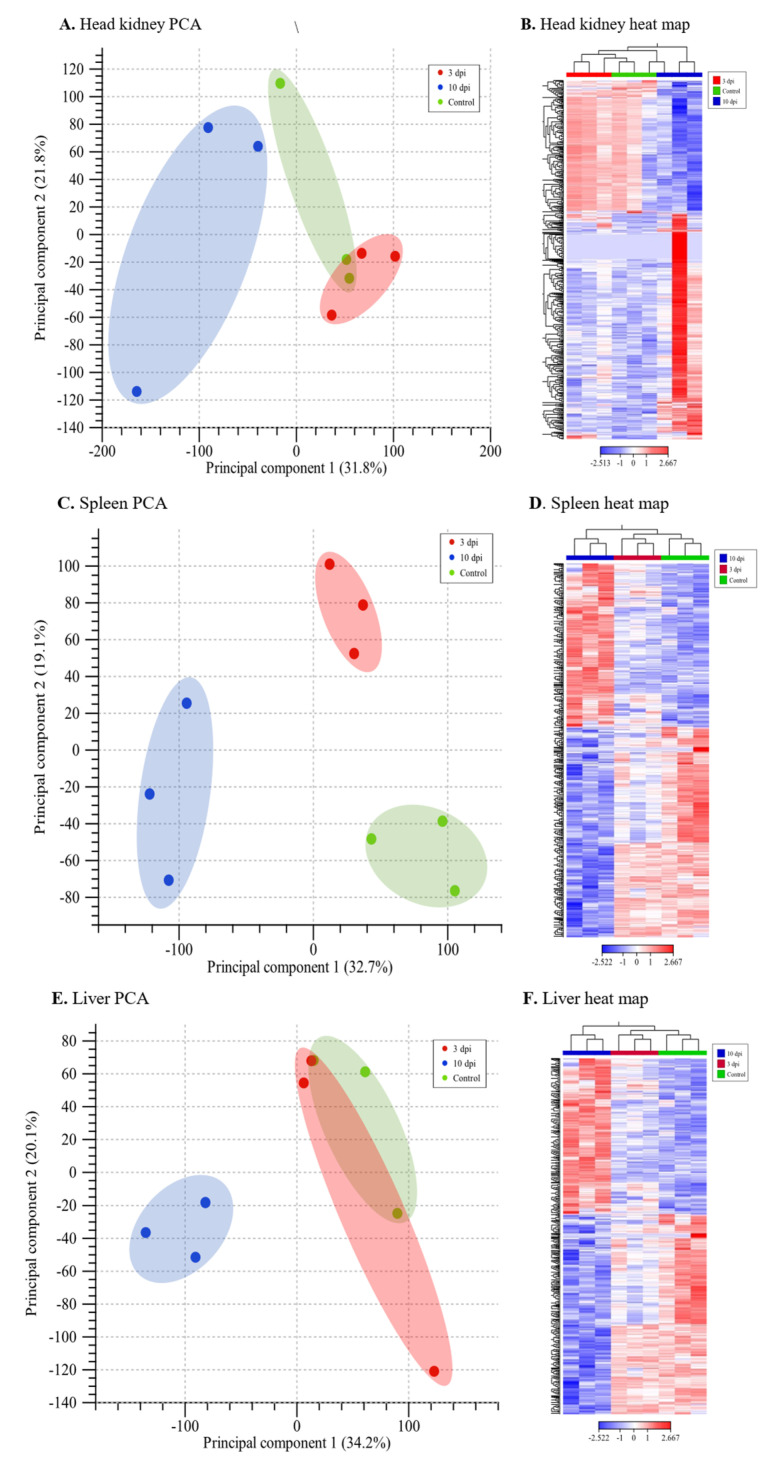
**Clusterization of gene expression profile in different lumpfish organs infected *A. salmonicida*.** (**A**). Principal component analysis (PCA) of lumpfish head-kidney infected with *A. salmonicida,* green dot represents three control samples, red dot represents three 3 dpi samples, blue dot represents three 10 dpi samples; (**B**). Heatmap of lumpfish head-kidney infected with *A. salmonicida*; (**C**). PCA of lumpfish spleen infected with *A. salmonicida,* green dot represents three control samples, red dot represents three 3 dpi samples, blue dot represents three 10 dpi samples; (**D**). Heat map of lumpfish spleen infected with *A. salmonicida*; (**E**). PCA of lumpfish liver infected with *A. salmonicida,* green dot represents three control samples, red dot represents three 3 dpi samples, blue dot represents three 10 dpi samples; (**F**). Heat map of lumpfish liver and liver infected with *A. salmonicida*.

To study the lumpfish response to *A. salmonicida* infection, we profiled the global gene expression of the head-kidney, spleen, and liver at 3 and 10 dpi, compared to non-infected fish using RNA-Seq. A global gene expression correlation analysis showed a high degree of correlation (R^2^ = 0.89 to 0.98; *p* < 0.0001) between different experimental conditions (Figure S3). Principal component analysis (PCA) and heatmap results reveal a clear tissue and time point clusterization (Figure 2 and Figure 3). Among all the organs studied, the spleen has the clearest clusterization (Figure 3).

**Figure 4:**
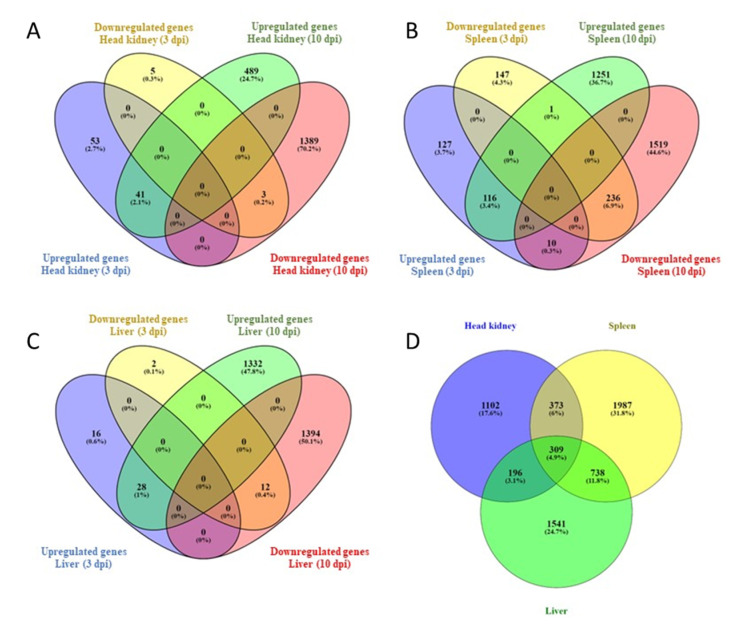
Gene expression profile comparison. (**A**). Venn diagram of upregulated and downregulated DEGs (log_2_FC ≥ 1, FDR ≤ 0.05) in head-kidney at 3 and 10 dpi; (**B**). Venn diagram of upregulated and downregulated DEGs (log_2_FC ≥ 1, FDR ≤ 0.05) in spleen at 3 and 10 dpi; (**C**). Venn diagram of upregulated and downregulated DEGs (log_2_FC ≥ 1, FDR ≤ 0.05) in the liver at 3 and 10 dpi; (**D**). Venn diagram of all DEGs (log_2_FC ≥ 1, FDR ≤ 0.05) in head-kidney, spleen, and liver.

The log_2_ fold-change (FC) ≥ 1 and false discovery rate (FDR) *p*-value of ≤0.05 were set as the cut-off criteria for sorting out significant differentially expressed genes. We found 102 differentially expressed genes (DEGs) in the head-kidney at 3 dpi. These DEGs included 94 upregulated and 8 down-regulated genes (Table 1, Figure 4A). Also, 1922 DEGs were identified in the head-kidney at 10 dpi. These DEGs included 530 upregulated and 1392 downregulated genes (Table 1, Figure 4A). In the spleen, 637 DEGs were identified at 3 dpi, including 253 upregulated and 384 downregulated genes (Table 1, Figure 4B). In the spleen, 3133 DEGs were identified at 10 dpi, including1368 upregulated and 1765 down-regulated genes (Table 1, Figure 4B). In the liver, 58 DEGs were identified at 3 dpi. These DEGs included 44 upregulated and 14 downregulated genes (Table 1, Figure 4C). Also, 2766 DEGs were identified in the liver at 10 dpi, including 1360 upregulated and 1406 downregulated genes (Table 1, Figure 4C). Gene identifier, description/annotation, fold-change, and FDR (*p*-value) are listed in File S1. A comparison between the head-kidney, spleen, and liver, including 3- and 10- dpi, showed that 309 DEGs were common to all organs, while the head-kidney and the spleen shared 373 DEGs, the head-kidney and the liver shared 196 DEGs, and the spleen and the liver shared 738 DEGs (Figure 4D).

Similarly, the log_2_FC ≥ 1 and FDR *p*-value of ≤0.05 were set as the cut-off criteria for sorting out significant differentially expressed transcripts (DETs). We identified 133 DETs in the head-kidney at 3 dpi, including 89 upregulated and 44 down-regulated transcripts (Table 2). Also, 699 DETs were identified in the head-kidney at 10 dpi, including 451 upregulated and 248 downregulated transcripts (Table 2). In the spleen, 614 DETs were identified at 3 dpi, including 237 upregulated and 377 downregulated transcripts (Table 2). In the head-kidney, 2152 DETs were identified at 10 dpi, including 1173 upregulated and 979 down-regulated transcripts (Table 2). In the liver, 33 DETs were identified at 3 dpi, including 27 upregulated and six downregulated genes (Table 2). Also, 1697 DETs were identified in the liver at 10 dpi, including 887 upregulated and 810 downregulated transcripts (Table 2). Gene identifier, fold-change, and FDR (*p*-value) are listed in File S2.

### 3.4. Global Profile of Differentially Expressed Transcripts Identified by De Novo Transcriptome 

To identify potential novel genes and transcripts, a *de novo* transcriptome analysis was conducted. Quality filtering and trimming were performed by trimmomatic, and approximately 4.28% of the raw reads were removed (Table S4). The remaining high-quality reads (originating from the three different lymphoid tissues) were used to build a *de novo* transcriptome assembly using Trinity v2.8.4 assembler.

The *de novo* assembly resulted in 403,204 transcripts with an average read length of 497 bp, representing 270,150 genes identified by Trinity (Table S4). The total length of all assembled transcripts is 522,614,427 bp with an N50 length of 3235 bp and GC content of 45.99%. We found that more than 98% of the reads were successfully aligned consistently for each sample (Table S5). Coding transcripts assessment was performed using the blastx search program in the database NCBI, RefSeq RNA, and SwissProt [71,72]. 

We further evaluated the completeness of the transcriptome assembly using BUSCO. Busco pipeline for gene set completeness was assessed for eukaryotes (*n* = 303), vertebrates (*n* = 2586), and actinopterygian (*n* = 4584). The analysis reported that the majority of the actinopterygian core genes had been successfully recovered from the lumpfish *de novo* assembly. Specifically, of the 4584 single-copy orthologs searched, ~88% were completely recovered, and ~4% were partially recovered. Only ~8% of single-copy orthologs were classified as missing in the assembly. This data indicates a complete, consistent, high-quality lumpfish transcriptome assembly (Figure S4). 

DETs identified from the three lymphoid tissues at different time points are summarized in File S3. The lists of DETs identified by DESeq2 were generally higher and were almost accommodated within the edgeR DETs lists. We used the more conservative edgeR-generated DETs for further analysis (Table 3). The log_2_ fold-change (FC) ≥ 1 and FDR *p*-value of ≤0.05 were set as the cut-off criteria for sorting out significant differentially expressed transcripts.

We found 286 DETs in the head-kidney 3 dpi, including 138 upregulated and 148 down-regulated transcripts (Table 3, Figure S5A). Also, 477 DETs were identified in the head-kidney at 10 dpi, including 204 upregulated and 273 down-regulated transcripts (Table 3, Figure S5A). In the spleen, 501 DETs were identified at 3 dpi, including 214 upregulated and 287 down-regulated transcripts (Table 3, Figure S5B). Also in the spleen, 2415 DETs were identified at 10 dpi, including 1005 upregulated and 1410 downregulated transcripts (Table 3, Figure S5B). In the liver, 133 DETs were identified at 3 dpi, including 56 upregulated and 77 downregulated transcripts (Table 3, Figure S5C). Also in the liver, 2093 DETs were identified at 10 dpi, including 1053 upregulated and 1040 downregulated transcripts (Table 3, Figure S5C). A comparison between all-time points of the head-kidney, spleen, and liver showed 56 DETs in common, while the head-kidney and spleen shared 53 DETs, the head-kidney and liver shared 26 DETs, and the spleen and liver shared 274 DETs (Figure S5D).

The hierarchal cluster of DETs expressed in abundance (log_2_FC ≥ ±5 and FDR ≤ 0.05) visualized as in the heatmap supports the tissue and time point specific clustering (Figure S6A). The heatmap also reveals that samples from uninfected lumpfish and infected animals clustered mostly within each tissue sub-cluster (Figure S6A). Also, we observed that the transcriptomic response was clearly separated based on infection time points in the spleen (Figure S6A). On the other hand, transcript responses in head-kidney and liver samples from the pre-infected fish and infected fish at 3 dpi were not highly differentiated, indicating an early process of infection in these tissues (Figure S6A). We also assessed and visualized inter and intragroup variability using Pearson’s correlation plots of correlation values between samples that agree with the hierarchal clustering analysis (Figure S6B). 

**Figure 5:**
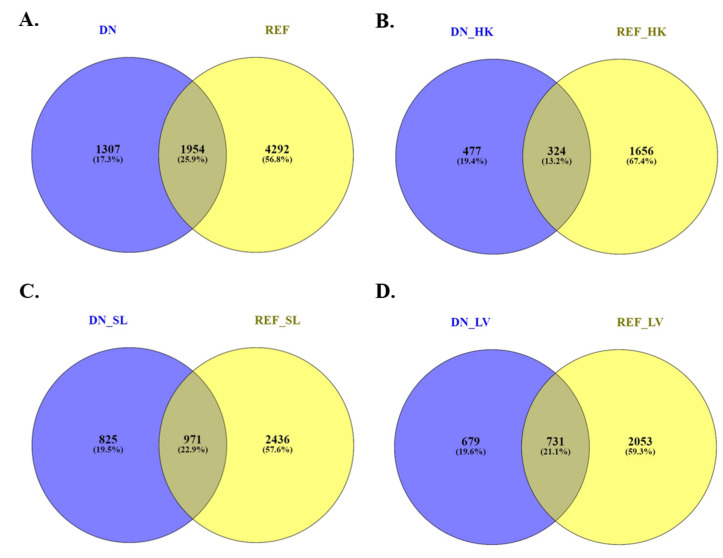
**Comparison of DEGs identified by reference genome-guided and *de novo* transcriptome assembly.** (**A**). Comparison of DEGs identified by reference genome-guided and *de novo* transcriptome assembly; (**B**). Comparison of DEGs identified by reference genome-guided and *de novo* transcriptome assembly in the head-kidney; (**C**). Comparison of DEGs identified by reference genome-guided and *de novo* transcriptome assembly in the spleen; (**D**). Comparison of DEGs identified by reference genome-guided and *de novo* transcriptome assembly in the liver; REF: reference genome-guided transcriptome assembly. DN: *de novo* transcriptome assembly. HK: Head-kidney. SL: Spleen. LV: Liver. DEGs: differentially expressed genes. Filtration of DEGs is log_2_FC ≥ 1, FDR ≤ 0.05.

Furthermore, a blastn analysis of all DETs identified by *de novo* assembly was conducted against the lumpfish genome using Blast+ 2.12.0 to retrieve lumpfish gene symbols corresponding to those transcripts (File S4). The analysis identified 1954 genes that were common to the DEGs identified by the reference genome-guided transcriptome analysis. In total, 1307 unique genes were identified, which included 477 genes in the head-kidney, 825 genes in the spleen, and 679 genes in the liver (Figure 5 and File S5). These unique genes were added to the DEGs list generated by the reference genome-guided transcriptome for GO enrichment analysis.

### 3.5. Gene Ontology Enrichment Analysis

Overall, the GO enrichment analysis using a combination of all DEGs at 3 dpi and 10 dpi identified multiple enriched GO terms related to biological process (BP), molecular function (MF), and cellular component (CC) (Figures S7 and S8, and File S6). This result suggests that nucleic acid metabolism and immune responses are mostly affected at the early point of infection. On the other hand, a lethal *A. salmonicida* infection could modulate lumpfish adaptive immune responses and metabolic processes.

**Figure 6:**
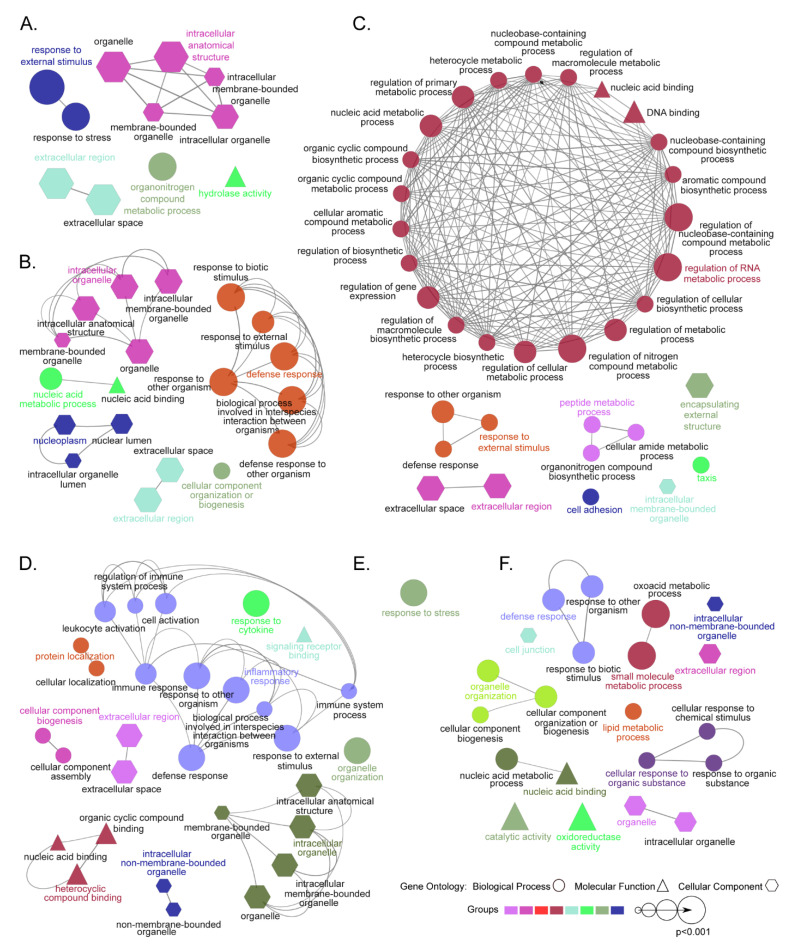
**ClueGO-based enriched gene ontology (GO) terms in lumpfish lymphoid organs.** (**A**). GO terms identified from DEGs in the head-kidney at 3 dpi; (**B**). GO terms identified from top 600 DEGs in the head-kidney at 10 dpi; (**C**). GO terms identified from DEGs in the spleen at 3 dpi; (**D**). GO terms identified from top 600 DEGs in spleen at 10 dpi; (**E**). GO terms identified from DEGs in liver at 3 dpi; (**F**). GO terms identified from top 600 DEGs in liver at 10 dpi. The size shows the GO term significance (the bigger the size the higher the significance). The shapes depict the database source i.e., GO biological process (ellipse), GO cellular component (hexagonal), and GO molecular function (triangles). The statistics of representative GO terms or pathways are tabulated in File S6.

The GO enrichment analysis using all DEGs in the head-kidney at 3 dpi identified GO terms associated with BP (e.g., response to stimulus), MF (e.g., hydrolase activity), and CC (e.g., intracellular anatomical structure) (Figure 6A, File S6). The upregulated DEGs in the head-kidney at 3 dpi were associated with acute phase response, inflammatory response, complement activation, negative regulation of immune effector process, fibrin clot formation and others (File S7). However, no GO terms were enriched by the downregulated DEGs of the head-kidney at 3 dpi. All the DEGs of the spleen at 3 dpi showed their association with several enriched GO terms related to BP (e.g., cell adhesion, defense response, nucleic acid metabolic process), MF (DNA binding), and CC (e.g., extracellular region, external encapsulating structure) (Figure 6C, File S6). Upregulated DEGs were mostly associated with acute phase response, complement component activation, humoral immune response, inflammatory responses, and many others (File S7). Downregulated DEGs of the spleen at 3 dpi were associated with ribosome assembly, cytoplasmic translation, and oxygen carrier (File S7). Furthermore, the DEGs of the liver at 3 dpi were only associated with response to stress (Figure 6E and File S6). Upregulated DEGs showed three enriched GO terms, such as acute phase response, chemoattractant activity, and cellular response to interleukin-1 (File S7), and downregulated DEGs of the liver at 3 dpi were not associated with any GO terms.

Furthermore, the 600 most significant DEGs (lowest FDR *p*-value) in the head-kidney at 10 dpi were associated with eight enriched GO terms related to BP (e.g., defense response, nucleic acid metabolic process), 1 GO term related to MF (nucleic acid binding), and 10 GO terms related to CC (e.g., organelle, nucleoplasm, extracellular region) (Figure 6B and File S6). The 600 most significant DEGs (lowest FDR *p*-value) of the spleen at 10 dpi were associated with 16 enriched GO terms related to BP (e.g., immune response, inflammatory response, defense response), four GO terms associated with MF (signaling receptor binding, organic cyclic compound binding, heterocyclic compound binding, and nucleic acid binding), and nine GO terms associated with CC (e.g., organelle, extracellular region) (Figure 6D, File S6). The 600 most significant DEGs of the liver at 10 dpi were associated with 13 enriched GO terms related to BP (e.g., defense response, small molecule metabolic process, lipid metabolic process, and nucleic acid metabolic process), three GO terms related to MF (catalytic activity, nucleic acid binding, and oxidoreductase activity), and five GO terms related to CC (e.g., organelle, cell junction, extracellular region) (Figure 6F and File S6). 

Furthermore, our GO enrichment analysis indicates that upregulated DEGs of the head-kidney at 10 dpi were associated with acute phase response, complement activation, inflammation, regulation of the apoptosis process, and negative regulation of the immune effector process (File S7). Upregulated DEGs in the spleen at 10 dpi were associated with complement activation, regulation of the apoptotic process, acute-phase response, blood coagulation, and inflammatory response (File S7). Upregulated DEGs of the liver at 10 dpi were associated with acute-phase response and inflammatory response (File S7).

Downregulated DEGs of the head-kidney at 10 dpi were associated with metabolic processes, ion transport, and microtubule bundle formation (File S7). Downregulated DEGs in the spleen at 10 dpi were associated with cytoskeleton organization, nucleic acid metabolic process, ribosome biosynthesis, and translation (File S7). Downregulated DEGs of the liver at 10 dpi were associated with metabolic processes, such as lipid, organic acid, amino acid, DNA, and RNA metabolic process, ion transport, DNA repair, double-strand break repair, and cell cycle (File S7).

### 3.6. Analysis of the Most Significant DEGs

**Figure 7:**
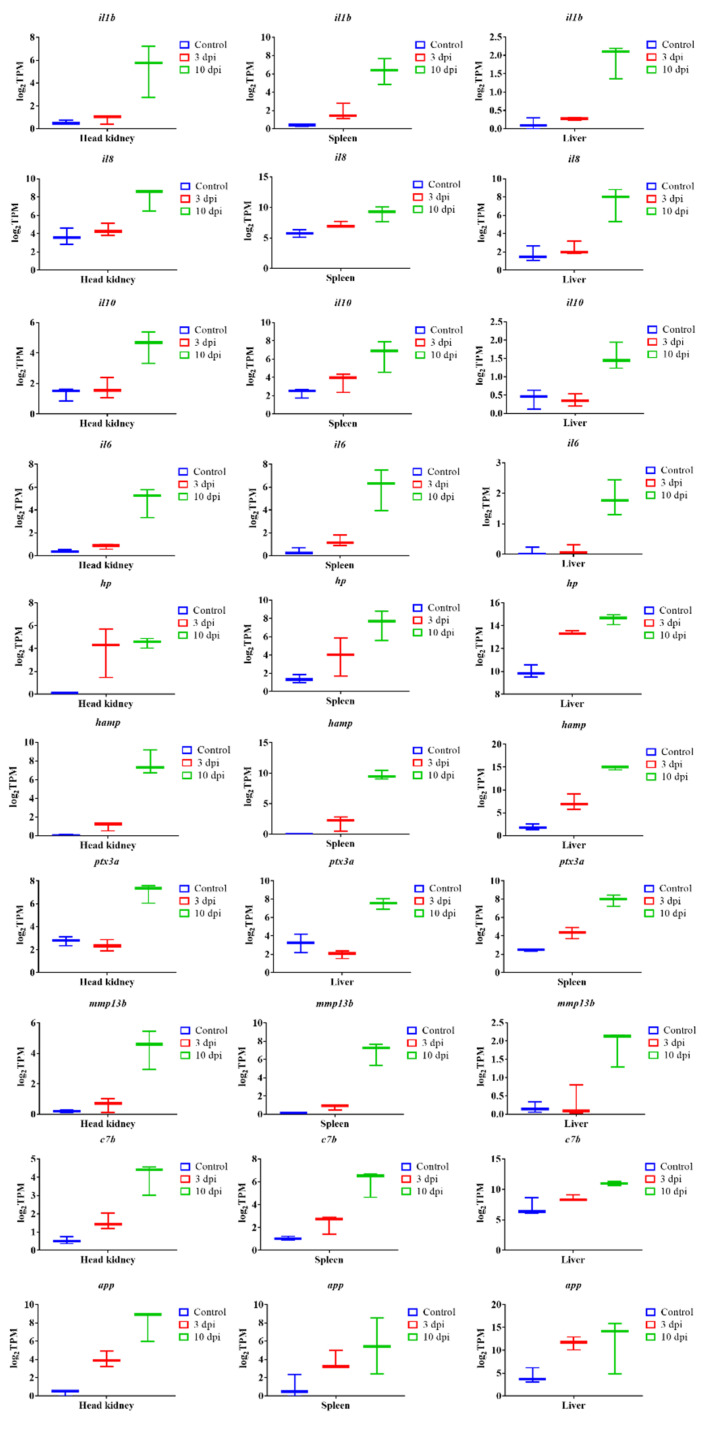
**Most significant DEGs in all organs studied after *A. salmonicida* infection in lumpfish.** Bar plots represent the expression pattern (log_2_TPM) of *interleukin-1 beta (il1b), interleukin-8 (il8), interleukin-10 (il10), interleukin-6 (il6), haptoglobin (hp), hepcidin (hamp), pentraxin-related protein PTX3 (ptx3), collagenase (mmp13b), complement component C7 (c7), and amyloid protein A (app)*.

**Figure 8:**
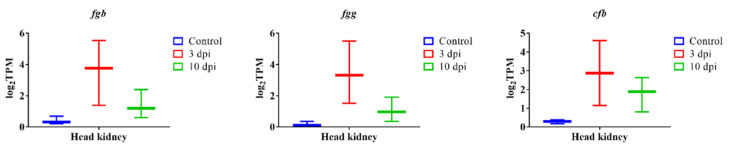
**Most significantDEGs in the head-kidney after *A. salmonicida* infection in lumpfish.** Bar plots represent the expression pattern (log_2_TPM) of *fibrinogen beta and gamma chain (fgb and fgg), complement factor B (cfb)*.

**Figure 9:**
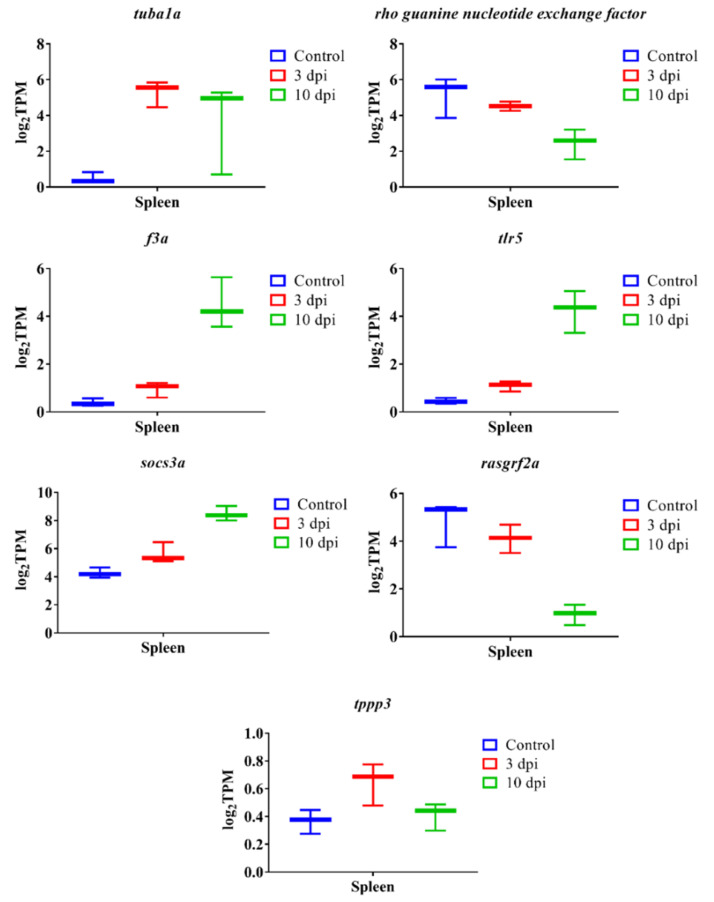
**Most significantDEGs in the spleen after *A. salmonicida* infection in lumpfish.** Bar plots represent the expression pattern (log_2_TPM) of *tubulin alpha-1A chain (tuba1a), rho guanine nucleotide exchange factor, coagulation factor IIIa (f3a), toll-like receptor 5 (tlr5), suppressor of cytokine signaling 3a (socs3a), ras-specific guanine nucleotide-releasing factor 2a (rasgrf2a), protein family member 3 (tppp3)*.

**Figure 10:**
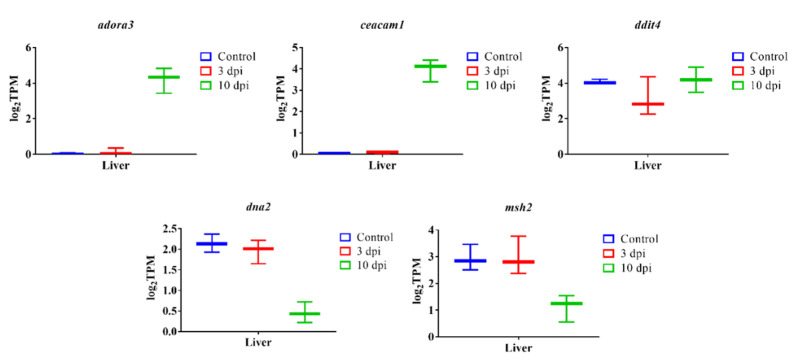
**Most significantDEGs in the liver after *A. salmonicida* infection in lumpfish.** Bar plots represent the expression pattern (log_2_TPM) of *adenosine receptor A3 (adora3), carcinoembryonic antigen-related cell adhesion molecule 1 (ceacam1), DNA damage-inducible transcript 4 protein (ddit4), DNA replication ATP-dependent helicase/nuclease DNA2 (dna2), and DNA mismatch repair protein Msh2 (msh2)*.

We identified the 300 most significant DEGs based on the lowest FDR *p*-value in lumpfish head-kidney, spleen and liver (File S9). Our results indicate that the most significantly overexpressed genes in the head-kidney, spleen, and liver were *il1b*, *il8*, *il10*, *il6*, *hamp*, *haptoglobin* (*hp*), *ptx3*, *collagenase* (*mmp13b*), *c7b*, and *app* (Figure 7). In addition to this, the top significant upregulated genes in the head-kidney at 3 dpi were *fibrinogen beta and gamma chain* (*fbb and fbg*) and *complement factor B* (*cfb*) (Figure 8). The top significant upregulated gene in the spleen at 3 dpi was *tubulin alpha-1A chain (tuba1a)*, and at 10 dpi were *tlr5*, *coagulation factor IIIa* (*f3*), and *socs3a* (Figure 9). The top significant upregulated genes in the liver at 10 dpi were *adenosine receptor A3* (*adora3*) and *carcinoembryonic antigen-related cell adhesion molecule 1* (*ceacam1*) (Figure 10). Most of these genes are involved with inflammation, complement activation, blood coagulation, and acute phage responses. 

Furthermore, we observed significant downregulation of genes encoding MHCII and IgM in all analyzed organs (Table 4). In addition, *cd79a*, *cd79b*, and *cd209* were downregulated in the head-kidney, spleen, and liver (Table 4).

Additionally, host genes are associated with cytoskeleton organization (e.g. *actin-binding LIM protein 1-like, cdc42 effector protein 1b, rho GTPase-activating protein 4-like, rho guanine nucleotide exchange factor 10-like protein, rho guanine nucleotide exchange factor 18, rho-related GTP-binding protein RhoH, tubulin beta chain, tubulin monoglycylase TTLL3-like, tubulin polyglutamylase TTLL7, ras-specific guanine nucleotide-releasing factor 2a (rasgrf2a), protein family member 3 (tppp3)*) were downregulated in the spleen at 10 dpi (Figure 9, Files S1, S7, and S8). 

*A. salmonicida* infection upregulated several regulators of NFκB activity, including *the inhibitor of κB (IκB), B-cell lymphoma 3 (bcl-3), tumor necrosis factor receptor superfamily member 11B (tnfrsf11b), apoptosis-enhancing nuclease (aen), DNA damage-inducible transcript 4 protein (ddit4), nfκb inhibitor α (nfkbiα), and nuclear factor interleukin-3-regulated protein (nfil3)* in lumpfish head-kidney and spleen (Files S1 and S8).

*A. salmonicida* infection downregulated several genes (e.g. *BRCA1-associated RING domain protein 1 (bard1), DNA replication ATP-dependent helicase/nuclease DNA2 (dna2), DNA excision repair protein ERCC-1 (ercc1), DNA repair endonuclease XPF (ercc4), E3 ubiquitin-protein ligase HERC2 isoform X1 (herc2), DNA mismatch repair protein Msh2 (msh2), Poly(ADP-ribose) polymerase 1 (parp1), DNA repair protein RAD52 homolog isoform X1 (rad52), DNA repair and recombination protein RAD54-like (rad54l)*) involved in DNA damage repair in liver 10 dpi (Figure 10, Files S1, S7, and S8). 

### 3.7. qPCR Verification Analysis

**Figure 11:**
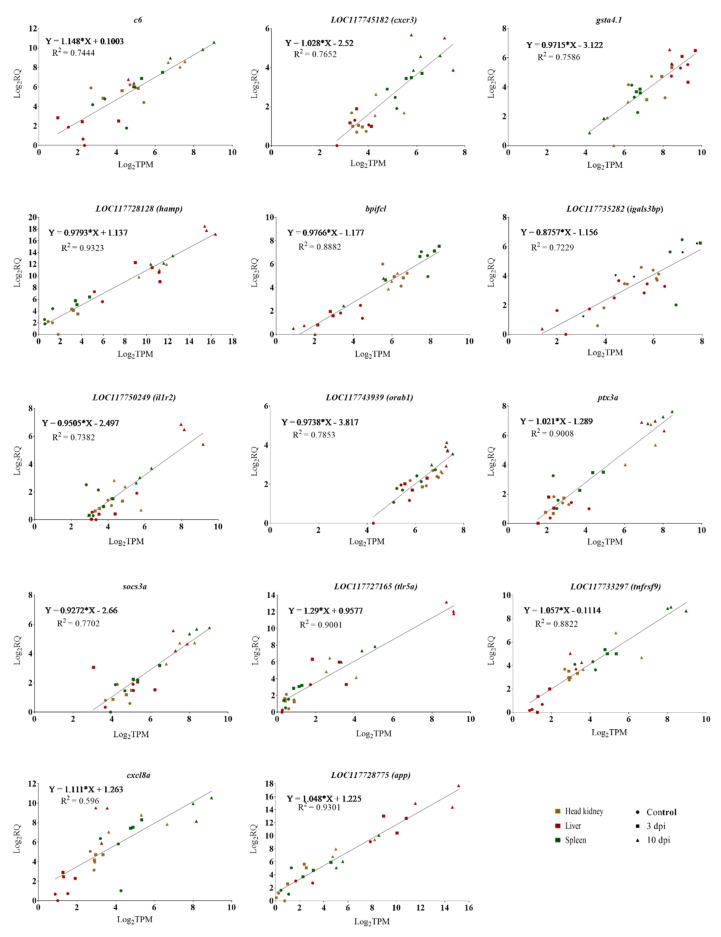
**Gene expression correlation between RT-qPCR and RNA-Seq data of 14 selected DEGs**. RNA-Seq data are presented as log_2_TPM (X-axis), and RT-qPCR data are represented as log_2_RQ (Y-axis). Three different colours represent gene expression in the head-kidney (brown), spleen (red) and liver (green). The circle represents control samples; the square represents 3 dpi, and the triangle represents 10 dpi. Each symbol is an average of three fish at a particular time point in that tissue.

The gene expression relationship between the log_2_ of the RQ values from the RT-qPCR and the log_2_ of the transcript per million reads (TPM) values from the RNA-Seq was determined for 14 selected genes, including *complement component c6 (c6), cxc chemokine receptor type 3 (cxcr3), galectin-3-binding protein a (igals3bp), glutathione s-transferase alpha tandem duplicate 1 (gsta4.1), hepcidine (hamp), interleukin 1 receptoe 2 (il1r2), interleukin 8 (cxcl8b/il8), bactericidal permeability-increasing protein (bpifcl), pentraxin-related protein ptx3 (ptx3a), ras-related protein orab-1 (orab1), amyloid protein a (app), suppressor of cytokine signaling 3a (socs3a), tumor necrosis factor receptor superfamily member 9 (tnfrsf9), and toll-like receptor 5 (tlr5a)*. As shown in Figure 11, there was a significant positive correlation between the RNA-Seq and RT-qPCR data. Correlation r^2^ values ranged from 0.6 to 0.93 for all 14 genes. These results indicate that all examined DEGs were in agreement with the reference genome-guided RNA-Seq analysis. On the other hand, the qRT-PCR results for *c6*, *bpifcl*, *igals3bp*, and orab1 were not in good agreement with the *de novo* RNA-Seq analysis (Figure S9). However, the qRT-PCR results of all other DEGs evaluated agreed with the *de novo* RNA-Seq analysis, with correlation R^2^ values ranging from 0.6 to 0.95 for the other 10 genes (Figure S9). 

## 4. Discussion

Lumpfish is an emergent cleaner fish species in the North Atlantic region. However, diseases, including bacterial diseases, are affecting the performance of lumpfish and its extended utilization. *A. salmonicida* is a globally distributed pathogen that infects and kills lumpfish [3]. The infection kinetics of *A. salmonicida* in lumpfish and its response to early and lethal infection has not been described. In this study, we established a reproducible *A. salmonicida* systemic infection model in lumpfish. Additionally, we examined the transcriptome profile of internal organs, including the head-kidney, spleen, and liver of lumpfish injected with a lethal dose (10^4^ CFU dose^−1^) of *A. salmonicida*, during early (3 dpi) and late infection stages (10 dpi). Head-kidney is known as a primary lymphoid organ as it is a hematopoietic tissue in the teleost, similar to the bone marrow of higher vertebrates [73]. B cell development, antigen-sampling and antigen retention have been described in teleost head-kidney [24,73,74]. The spleen is the primordial secondary lymphoid organ that contains macrophages, MHC class II+ cells, and T cells [24,73,75,76]. The liver is also an important organ that takes part in metabolism and defense [25,26,28,29], and it is also considered as a lymphoid organ, as non-parenchymal cells of the liver take part in antigen presentation and immunomodulatory functions. In addition, the liver encompasses large populations of natural killer cells and T cells [26,28]. This study analyzed the transcriptome response of the three main lymphoid tissues (head-kidney, spleen, and liver) of lumpfish during a lethal *A. salmonicida* infection.

The virulence of different *A. salmonicida* isolates varies in different fish hosts. For instance, *A. salmonicida* DH170821-10 showed relatively lower pathogenicity with an LD_50_ of 6.4 × 10^3^ CFU dose^−1^ in rainbow trout and coho salmon (*Oncorhynchus kisutch*) [77]. Another study described two highly pathogenic strains of *A. salmonicida*, MT1057, and MT423, with an LD_50_ of 10^2^ CFU dose^−1^ in Atlantic salmon but a lower virulence in halibut, with an LD_50_ of 10^6^ CFU dose^−1^ [78]. Our study showed that *A. salmonicida* J223 (Santander lab collection) is highly virulent for Newfoundland lumpfish. We determined that an ip infection of 10^2^ bacterial cells per dose can kill 50 percent of the infected lumpfish population, which is similar to rainbow trout (*Oncorhynchus mykiss*), Chinese perch (*Siniperca chuatsi*), and Atlantic salmon [78,79]. The hyper-virulence of *A. salmonicida* J223 strain in lumpfish was further verified by another study conducted by our group, where a bath infection of lumpfish with 10^6^ CFU mL^−1^ of *A. salmonicida* J223 caused 100% lethality within 14 dpi (unpublished data).

Subsequently, *A. salmonicida* infection kinetics in different organs was determined for different doses used to infect lumpfish. All lethal doses (10^3–^10^5^ CFU dose^−1^) showed the presence of *A. salmonicida* in the head-kidney at 3 dpi, suggesting that this organ is the primary *A. salmonicida* target, and from then it spreads to the spleen and liver, and finally, after 7 dpi, *A. salmonicida* infection in lumpfish becomes systemic (Figure 1). Similar to our findings, previous studies indicated that 3 to 4 days is a typical incubation period for *A. salmonicida*, where the bacterium rapidly disperses in the kidneys, followed by the spleen and liver [80,81]. Lumpfish infected with a low dose of *A. salmonicida* (10^1^ CFU dose^−1^) established a persistent infection, as bacterial colonies were still detected after 30 dpi without causing mortalities. While *A. salmonicida* J223 strain lethal doses cause acute infections, in low doses it might cause chronic infections.

Similarly, *Pseudomonas aeruginosa* can cause both symptomatic acute and chronic infections. While acute infections often spread rapidly and can damage tissues as well as contribute to high mortality by sepsis, chronic infections can be carried on for years [82]. We did not explore the further mechanism of *A. salmonicida* mediated chronic infection here. Future studies to consider how *A. salmonicida* can utilize strategies to evade immune clearance to cause chronic infections would be helpful to explore the pathogenesis in marine teleosts.

To understand the transcriptome dynamics and their impact on gene expression levels, high-throughput RNA-Seq technology was used. RNA-Seq can effectively analyze transcript sequences and estimate gene expression levels that can be applied to the identification of DETs or DEGs between different experimental conditions [83,84]. RNA-Seq results in millions of short reads which need to be assembled into transcript sequences [85]. An RNA-Seq analysis allows for the distinguishing between individual transcripts (isoform) of a gene [85]. Analysis of DETs is essential in identifying differences between tissues [84]. However, the alignment of RNA-Seq reads to a certain gene allows researchers to study gene expression [86,87]. Gene expression estimation from the expression levels of transcripts provides more robust results [88]. Gene expression estimation allows researchers to determine DEGs under different conditions. Analyzing DEGs is more applicable for biological analysis, e.g., GO enrichment analysis [89]. Our study utilized two different approaches: *de novo* and reference-based, to assemble the transcriptome. With the availability of the reference genome, a reference-based assembly is more effective than a *de novo* assembly [90]; however, studies showed that the *de novo* assemblies were able to identify a complete gene content [55,60,91,92,93,94,95]. We applied the *de novo* assembly approach at the isoform level that allowed us to determine DETs in 3 and 10 dpi of the head-kidney, spleen, and liver. However, the reference-based assembly approach allowed us to generate both DEGs and DETs using CLCGWB. Our results demonstrate that the total number of DETs identified by the *de novo* transcriptome assembly analysis was higher than the total number of DETs identified by the reference genome-guided transcriptome assembly analysis (5265 vs 4261, log_2_FC ≥ 1, FDR ≤ 0.05), which is similar to Kovi et al. [91] (Files S3 and S2). Intrinsic methodological issues of *de novo* analysis could generate misassembled transcripts [96]. The trinity *de novo* assembler might yield more transcripts because of lacking strand-specific information [97]. Subsequently, a BLAST search of all *de novo* DETs against the lumpfish genome identified that only 4839 (91.9%) *de novo* DETs are protein-coding transcripts. Hereafter, the corresponding genes of these *de novo* DETs were compared with the reference-based DEGs. We observed that only 25.9% of the genes were shared between the *de novo* and reference-based analysis, 17.3% of genes were unique in *de novo* analysis, and 56.8% genes were unique in reference-based analysis (Figure 5).

In addition to this, our qPCR verification analysis demonstrates that the overall gene expression levels were underestimated by *de novo* analysis (Figure 11 and Figure S9). Previous studies have found that the reference-based method surpasses the *de novo* method for characterizing transcriptome and gene expression [96,98,99]. Still, our study suggested that each method captured unique transcripts. Therefore, we adopted an integrative approach for GO enrichment analysis to bring more benefits for the better exploration of pathogenesis.

The number of DEGs and DETs was highest in the spleen, followed by the head-kidney and liver at 3 dpi. Similarly, the number of DEG and DET were highest in the spleen, followed by liver and head-kidney at 10 dpi. However, in most cases, the number of DETs were lower than that of DEGs. This is because the gene-level expression is global. One gene can have several transcripts as a result of alternative splicing in eukaryotes and not all the DETs were significant (log_2_FC ≥ 1, FDR ≤ 0.05). Therefore, we cannot compare between the gene and transcript expression. Thus, moving forward, we used the gene-level analysis.

The head-kidney plays a key role in initiating the immune response in fish [73,100]. We observed that the initial inflammatory response was triggered in the head-kidney at 3 dpi (File S8). The histopathological analysis also detected the highest level of inflammation in the head-kidney, followed by the spleen and liver, respectively (Figure 1F). Such responses correlate with the infection kinetics of *A. salmonicida* (Figure 1B–E). Nevertheless, the spleen also was infected very fast and showed a tremendous amount of DEGs and enriched GO terms (Figure 1C, Figure 4D and Figure 6C and Table 1). The spleen has a key role in promoting humoral immunity [101,102] and plays a key role in identifying cell damages [103]. This could be a reason for having the highest spleen response during *A. salmonicida* infection. The liver controls biochemical processes, including metabolism [104]. A metabolic arrest was suggested at 10 dpi in the liver (Files S6, S7 and S8). Interestingly, we observed fish lethargy (e.g., lack of appetite and swimming) starting at 7 dpi and continuing until death, which might relate to the metabolic arrest at the deadly point of the infection.

Our RNA-Seq analysis suggests that the most significantly upregulated genes are associated with inflammation, complement activation, blood coagulation, and acute phase responses (Figure 7, Figure 8, Figure 9 and Figure 10). Furthermore, GO enrichment analysis indicates that inflammation and acute phase response were enriched pathways in all three organs (Files S7 and S8). In addition, blood coagulation and complement activation were enriched in the head-kidney and spleen (File S7). Inflammation is an immune defense mechanism in response to bacterial infection where leukocytes (neutrophils and monocytes/macrophages) secrete cytokines into the bloodstream. Such cytokines, like IL1 and IL6, stimulate hepatocytes to produce and secrete acute phase proteins (APPs, e.g., serum amyloid proteins (SAPs), haptoglobin (HP)) [105,106,107]. RNA-Seq results demonstrate the upregulation of genes related to inflammation and acute phage responses in lumpfish head-kidney (e.g., *il1b*, *il6*, *il10*, *cxcl8a*, *serum amyloid A-3*, *hp*, *cxcr3*, *hamp*, *ptx3a*, *tlr5a*), spleen (e.g., *app*, *C-C motif chemokine 19 (ccl19)*, *il1b*, *il6*, *cxcl3*, *cxcl8a*, *hp*, *cxcr3*, *hamp*, *ptx3a*, *tlr5a*), and liver (e.g., *app*, *saa3*, *ccl19*, *il1b*, *il6*, *cxcl8a*, *hp*, *cxcr3*, *hamp*, *ptx3a*, *tlr5a*) (Files S7 and S8). The upregulation of several of these genes (*cxcr3*, *hamp*, *il1r2*, *cxcl8b/il8*, *ptx3a*, *app*, *tlr5a*) were further verified by the qPCR experiment (Figure 11). Like lumpfish, *A. salmonicida* infection also induces inflammation and acute phase response in Atlantic salmon, cod, rainbow trout, Arctic charr, and zebrafish [13,15,32,81,108,109]. The blood coagulation system and complement cascade are closely linked to the inflammatory response and acute-phase response [110,111,112]. Upregulation of genes related to blood coagulation was observed in lumpfish head-kidney at 3 dpi (e.g., *fibrinogen*, *prothrombin*, *plasminogen*, *antithrombin-III*) and spleen at 10 dpi (e.g., *thrombomodulin*, *platelet glycoprotein 4*, *coagulation factor XIII*, *coagulation factor IIIa*, and *coagulation factor VIII*, *von Willebrand factor*) (Files S1, S7 and S8). Furthermore, after RNA-Seq data analysis, the upregulation of genes *complement factor H*, *complement factor B*, *c3-like complement component (c3)*, *c7*, *c8 alpha chain complement component (c8)*, *c1r-A subcomponent-like complement component*, and c6 were observed in the head-kidney and spleen (Files S1 and S8). A qPCR analysis confirmed the upregulation of *c6* in all three tissues. These results indicate that *A. salmonicida* infection may induce blood coagulation and complement activation in lumpfish, similar to observations made in zebrafish, Atlantic salmon, and Arctic charr infections with *A. salmonicida* [13,15,81].

However, under some circumstances, these innate immune responses cause tissue damage and organ failure, eventually leading to death, which is a hallmark of sepsis [113]. During sepsis, the association of pattern recognition molecules with the pro-inflammatory mediators and activation of the NF-κB signaling cascade could cause the increased expression of proinflammatory cytokines [111]. Pro-inflammatory cytokines and complement components activate the coagulation cascade [114]. The coagulation system acts as a general host defense system to restrict the dissemination of pathogens by recruiting leukocytes, while fibrin promotes the adherence and migration of cells [115]. However, overactivation of the coagulation system during acute bacteremia causes disseminated intravascular coagulation (DIC), microvascular thrombosis–induced hypoxia, and a prolonged suppression of fibrinolysis, which contributes to multiorgan failure, abnormalities in host metabolism, immune suppression, septic like shock, and death [111,115,116,117,118]. Interestingly, the downregulation of genes encoding hemoglobin subunits alpha and beta was identified in the spleen at 3 dpi, and the moribund lumpfish was visually noticed with the symptoms of hypoxia (Files S1, S7, and S8).

The unrestricted activation of inflammation, blood coagulation, and complement systems break the blood/tissue barrier and damage the host tissue and organs [119]. Interestingly, excessive hemorrhages in the lumpfish body, eyes, gills, or at the base of the fins, muscles, and organ tissues and astics were visually observed in moribund fish (Figure S10). These observations suggest that detrimental and uncontrolled inflammation, overactivation of blood coagulation, and complement components might lead to a septic-like shock, which plays a significant role in the *A. salmonicida* mediated lethal infection of lumpfish. However, the septic response is an extremely complex reaction of inflammatory, anti-inflammatory, humoral, and cellular processes, and circulatory abnormalities, which are highly variable with the non-specific nature of the symptoms [120]. Therefore, further investigation is required to confirm sepsis in lumpfish.

Furthermore, *il10* was upregulated in all three lumpfish organs at 10 dpi (Figure 7). Previously, it was described that *A. salmonicida* elicits a significant increase in *il10* expression in head-kidney leucocytes [121]. A similar effect was also described in Arctic char [81]. IL-10 can contribute to the immune suppression by inducing a Treg-mediated response. Deleting the T3SS genes of *A. salmonicida* decreases the host cytokine expression significantly [121]. A fully virulent *A. salmonicida* downregulated specific innate and adaptive immune gene expression and reduced the survival of the infected rainbow trout [122,123,124]. Consequently, we observed the downregulation of genes encoding MHCII and IgM in all analyzed organs (Table 4). Previous research on *A. salmonicida* infection in trout showed the downregulation of immunoglobulin light chains, constant and variable domains [108,109]. In addition, *cd79a*, *cd79b*, and *cd209* were downregulated in the head-kidney, spleen, and liver (Table 4). CD79a and CD79b are B-cell antigen receptor complex-associated proteins α, and β chains play a crucial role in B cell development and antibody production [125]. CD209 is a C-type lectin, an essential PRR that participates in immune defense and microbial pathogenesis in mammals, and it is present on the surface of macrophages [126]. Coincident, previous studies on *A. salmonicida* infection in rainbow trout showed that *cd209* was downregulated [108,109]. All of these observations suggest the *A. salmonicida* mediated immune suppression in lumpfish.

*A. salmonicida* virulence factor AopO is an ortholog of the *Yersinia* YopO/YpkA serine/theonine kinase. This serine/threonine kinase inhibits phagocytosis by blocking the Rac-dependent Fcγ receptor internalization pathway [122]. We observed the downregulation of the low-affinity immunoglobulin gamma Fc region receptor II-b-like (*LOC117747925*) in the spleen at 3 dpi (File S1), suggesting that *A. salmonicida* J223 might cause an antiphagocytic effect in lumpfish. However, this could be the result of an effect of undetermined signaling cascade, which needs further verification.

At least six *A. salmonicida* type-3 secretion system-related virulence factors, AexT, Ati2, AopH, AopO, AopN, and AopS could be responsible for disrupting the host cytoskeleton structure, which allows this pathogen to colonize and survive inside the host [30,122]. The GTPase activating domain and the ADP-ribosylating domain of AexT act on small monomeric GTPases of the Rho family (Rho, Rac, and Cdc42) and actin, respectively, and causes actin depolymerization and cell rounding [30,122]. Ati2 of *Vibrio parahaemolyticus* is responsible for the local detachment of the actin-binding proteins from the plasma membrane and induces membrane blebbing and cytolysis by hydrolyzing the host phosphatidylinositol 4,5-bisphosphate [30,122]. AopH, an ortholog of *Yersinia* YopH, is responsible for altering the actin cytoskeleton by dephosphorylating the tyrosine residue [30,122]. AopO, an ortholog of *Yersinia* YopO, prevents the actin distribution in the host cell [30,122]. AopN, an *A. salmonicida* effector, binds and sequesters αβ-tubulin and inhibits microtubule polymerization that induces mitotic arrest [30,122]. AopS, an ortholog of *V. parahaemolyticus* VopS, could inhibit the actin assembly by preventing the interaction of Rho GTPases with its downstream effectors [30,122]. In this study, lumpfish DEGs associated with cytoskeleton organization (e.g. *actin-binding LIM protein 1-like, cdc42 effector protein 1b, rho GTPase-activating protein 4-like, rho guanine nucleotide exchange factor 10-like protein, rho guanine nucleotide exchange factor 18, rho-related GTP-binding protein RhoH, tubulin beta chain, tubulin monoglycylase TTLL3-like, tubulin polyglutamylase TTLL7, rasgrf2a, tppp3*) were downregulated in the spleen at 10 dpi (Figure 9 and Files S1, S7, and S8). In addition, downregulation of genes related to microtubule bundle formation was observed in the head-kidney at 10 dpi (e.g., genes encoding dynein assembly factors, dynein heavy chains, tppp3) Figure 9 and Files S1, S7, and S8). These findings indicate that the disruption of the lumpfish cytoskeleton might be possible by actin and microtubule depolymerization and mitotic arrest during *A. salmonicida* infection.

The *A. salmonicida* type-3 secretion system (T3SS) effector AopP induces apoptosis in affected cells by interfering with critical signal transduction pathways (i.e., NFκB signaling) that activate caspase 3 [30,122]. AopP hinders the NF-κB signaling pathway by restraining the transportation of the p50/p65 protein complex (NFKB1/RelA) into the target cell’s nucleus [30,122], resulting in the septicemia and furuncles formation (subcutaneous wounds) in host tissue [127]. We observed the upregulation of several genes that positively regulate the apoptosis process in the head-kidney and spleen at 10 dpi (Files S1, S7, and S8). However, no caspases were differentially expressed in this study. We detected the upregulation of several regulators of the NFκB pathway, including κB (IκB) inhibitor, *bcl-3, tnfrsf11b, aen, ddit4, nfkbia*, and *nuclear factor interleukin-3-regulated protein* (*nfil3*) (Files S1 and S8). We did not observe the formation of furuncles in lumpfish skin that might be concurrent with no expression of caspases involved in apoptosis. A dual transcriptomic study and future in vitro experiments to identify the dysregulation of *aopP* of *A. salmonicida* in lumpfish lymphoid organs could be valuable for future research. 

Certain bacterial pathogens could cause chronic inflammation and/or produce genotoxins that can damage proteins, lipids, metabolites, DNA, and RNA. For example, *Helicobacter pylori* infection downregulates DNA mismatch repair and base excision repair mechanisms [128]. The bacterial toxin can be a source of DNA double-strand breaks (DBSs), causing cell death [129]. DSBs induces DNA damage response (DDR), resulting in cell cycle arrest [130]. Our results indicate that *A. salmonicida* infection downregulates several genes involved in DNA damage/repair in the liver at 10 dpi (e.g., *bard1, dna2, ercc1, ercc4, herc2, msh2, parp1, rad52, rad54l*) (Figure 10 and Files S1, S7, and S8). Therefore, several biological processes such as DNA replication, DNA and RNA metabolic processes, double-strand break repair, DNA repair, RNA metabolic process, gene expression, and cell cycle processes were enriched by the downregulated genes in liver 10 dpi (File S7). These findings suggests that *A. salmonicida* infection might provoke lumpfish DNA damage and cause cell cycle arrest in lumpfish liver.

Suitable biomarkers of sepsis and infection are necessary for monitoring fish disease conditions [131]. *hamp*, *hp*, *app*, *ptx3*, *mmp13b*, *il1b*, *il8*, *il10*, and *il6* were significantly upregulated in the head-kidney, spleen, and liver of infected lumpfish, suggesting they could be used as biomarkers for the molecular diagnosis of *A. salmonicida* infection (Figure 7). Actually, most of these genes were suggested as biomarkers of sepsis in humans [132,133], suggesting a conserved response to septic shock in vertebrates. Genes encoding ras-related GTPase 1Ab, rho GTPase-related proteins, and microtubule-associated proteins might be proposed as biomarkers to identify *A. salmonicida* specific infection (Figure 9 and File S9). In addition, *tlr5*, *c6*, *c7*, *fgb*, *fgg*, *f3a*, *socs3a*, *adora3*, *ceacam1*, *tppp3*, *tuba1a*, *ddit4*, *dna2*, and *msh2* can also potentially be proposed as a biomarker to detect *A. salmonicida* lethal infection in lumpfish. Multiplex qPCR assays for these genes could then be developed to detect early *A. salmonicida* infection in lumpfish. These high-throughput technologies could accelerate the identification of potential biomarkers for various diagnostic and therapeutic developments in the future lumpfish aquaculture industry and explore the repose to septic shock in marine teleost.

## 5. Conclusions

**Figure 12:**
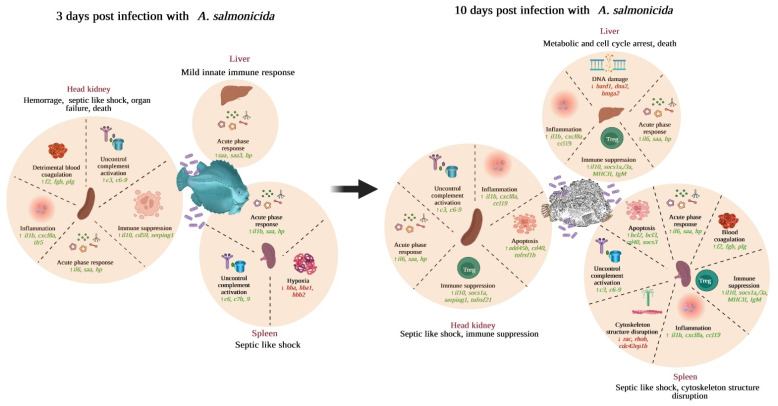
***Aeromonas salmonicida* infection model in lumpfish lymphoid organs.***A. salmonicida* early (3 dpi) and late (10 dpi) infection model suggests that *A. salmonicida* induces lethal infection in lumpfish by uncontrolled and detrimental blood coagulation, complement activation, and inflammation. Such responses lead to hypoxia, internal organ hemorrhages, suppression of the adaptive immune system, and impairment of the DNA repair system, which results in cell cycle arrest, and, ultimately, death. Furthermore, *A. salmonicida* could destabilizes the cytoskeleton structure by depolymerizing actin and microtubule.

*A. salmonicida* has evolved a myriad of mechanisms to counteract and modulate the host responses. Only 10^2^ cells of *A. salmonicida* can kill 50% of the lumpfish population. Overall, our study characterizes *A. salmonicida* infection kinetics in lumpfish head-kidney, spleen, and liver (Figure 1) and proposes an infection model for lumpfish molecular responses at the early and lethal point of infection (Figure 12). The model suggests that *A. salmonicida* might induce lethal infection in lumpfish by uncontrolled and detrimental blood coagulation, complement activation, and inflammation. Such responses could lead to hypoxia, internal organ hemorrhages, suppression of the adaptive immune system, and impairment of the DNA repair system, which results in cell cycle arrest, and, ultimately, death (Figure 12). Also, *A. salmonicida* might destabilize the cytoskeleton structure by depolymerizing actin and microtubules to colonize and survive inside the lumpfish (Figure 12). In addition, *A. salmonicida* may be capable of inhibiting the NF-kB signaling pathway and the induction of the apoptotic process (Figure 12). These findings could help a global understanding of the molecular network of *A. salmonicida*-lumpfish host interactions, which is essential for developing effective treatments. Furthermore, our analysis provides a guideline for future experimental designs to study *A. salmonicida* pathogenesis in lumpfish.

## Figures and Tables

**Table 1:** Differentially expressed Genes (DEGs) identified by reference transcriptome analysis.

Tissues	Days Post-Infection (dpi) with *A. salmonicida*	Upregulated Genes	Downregulated Genes	Total Genes
Head-kidney	3	94	8	102
Head-kidney	10	530	1392	1922
Spleen	3	253	384	637
Spleen	10	1368	1765	3133
Liver	3	44	14	58
Liver	10	1360	1406	2766

**Table 2:** Differentially expressed transcripts (DET) identified by reference transcriptome analysis.

Tissues	Days Post-Infection (dpi) with *A. salmonicida*	Upregulated Transcripts	Downregulated Transcripts	Total Transcripts
Head-kidney	3	89	44	133
Head-kidney	10	451	248	699
Spleen	3	237	377	614
Spleen	10	1173	979	2152
Liver	3	27	6	33
Liver	10	887	810	1697

**Table 3:** Differentially expressed transcripts (DET) identified by *de novo* transcriptome analysis.

Tissues	Days Post-Infection (dpi)	edgeR Upregulated Transcripts	edgeR Downregulated Transcripts	Total Transcripts
Head-kidney	3	138	148	286
Head-kidney	10	204	273	477
Spleen	3	214	287	501
Spleen	10	1005	1410	2415
Liver	3	56	77	133
Liver	10	1053	1040	2093

**Table 4:** Significant differential regulation of adaptive immune marker (log_2_FC ≥ 1, FDR ≤ 0.05).

Gene symbol	Description	Log_2_ Fold Change					
		Head-Kidney		Spleen		Liver	
		3 dpi	10 dpi	3 dpi	10 dpi	3 dpi	10 dpi
*LOC117737678*	H-2 class II histocompatibility antigen gamma chain	-	-	-	−1.91	−1.37	−1.68
*LOC117747618*	H-2 class II histocompatibility antigen, E-S beta chain	-	-	−1.12	−2.35	−1.58	−1.76
*LOC117731450*	H-2 class II histocompatibility antigen, A-U alpha chain	-	-	−1.03	−2.75	−1.16	−2.12
*LOC117747619*	H-2 class II histocompatibility antigen, A-U alpha chain	-	-	-	−2.79	−1.76	−1.65
*LOC117745568*	H-2 class II histocompatibility antigen, A-U alpha chain	-	1.23	-	-	-	−3.18
*LOC117742904*	V-set and immunoglobulin domain-containing protein 1	-	-	−2.20	−5.07	−1.92	−3.44
*si:ch211-139g16.8*	immunoglobulin superfamily member 6	-	-	-	−1.33	−1.04	−1.58
*Sema3fa*	Immunoglobulin (Ig)	-	−1.13	-	−1.81	-	−2.23
*vsig10*	V-set and immunoglobulin domain-containing protein 10	-	-	-	−1.85	-	−1.18
*LOC117747962*	immunoglobulin kappa light chain	-	-	-	−1.94	-	−1.34
*LOC117737972*	polymeric immunoglobulin receptor	-	-	-	−1.97	-	−1.68
*igsf8*	immunoglobulin superfamily member 8	-	-	-	−2.04	-	-
*igsf5b*	immunoglobulin superfamily member 5	-	−2.05	−1.64	−2.24	-	-
*tmigd1*	transmembrane and immunoglobulin domain containing 1	1.07	−4.07	−1.28	−2.56	-	-
*LOC117750007*	immunoglobulin-like domain-containing receptor 1	-	−2.14	−1.42	-	-	-
*vsig8b*	V-set and immunoglobulin domain containing 8b	-	−2.61	−1.94	-	-	-
*cd79a*	B-cell receptor complex-associated protein alpha	-	-	−2.27	−5.76	−1.33	−2.49
*cd79b*	B-cell receptor complex-associated protein beta	-	-	−1.21	−4.75	−1.87	−5.44
*LOC117735721*	CD209	−1.08	−1.69	-	−1.96	-	−2.53
*LOC117750415*	CD209	-	-	-	−2.17	−1.31	−3.50
*LOC117750406*	CD209	-	-	-	−2.90	-	−3.59

## Data Availability

Not applicable.

## Supplementary Materials

The following supporting information can be downloaded at: https://www.mdpi.com/article/10.3390/microorganisms10112113/s1, Figure S1. RNA-Seq sample collection and data analysis workflow. Figure S2. Quality of the RNA samples and sequenced reads. Figure S3. Global gene expression profile of different lumpfish organs infected *A. salmonicida*. Figure S4. Quality of transcriptome assembly. Figure S5. Transcript expression profile comparison. Figure S6. Correlation analysis, and hierarchical clustering. Figure S7. ClueGO-based enriched gene ontology (GO) terms in lumpfish lymphoid organs at 3 dpi with *A. salmonicida*. Figure S8. ClueGO-based enriched gene ontology (GO) terms in lumpfish lymphoid organs at 10 dpi with *A. salmonicida*. Figure S9. Gene expression correlation between RT-qPCR and RNA-Seq data of 14 gene expressions. Figure S10. *Aeromonas salmonicida* J223 infected lumpfish. Table S1. Quality of the RNA. Table S2. Trimming and mapping statistics. Table S3. Trinity statistics. Table S4. Alignment statistics. Table S5. Primers used in this study. File S1a. Differentially expressed genes (DEGs) in head-kidney 3 dpi identified by reference genome-guided transcriptome assembly (log_2_FC ≥ 1, FDR ≤ 0.05). File S1b. Differentially expressed genes (DEGs) in head-kidney 10 dpi identified by reference genome-guided transcriptome assembly (log_2_FC ≥ 1, FDR ≤ 0.05). File S1c. Differentially expressed genes (DEGs) in spleen 3 dpi identified by reference genome-guided transcriptome assembly (log_2_FC ≥ 1, FDR ≤ 0.05). File S1d. Differentially expressed genes (DEGs) in spleen 10 dpi identified by reference genome-guided transcriptome assembly (log_2_FC ≥ 1, FDR ≤ 0.05). File S1e. Differentially expressed genes (DEGs) in liver 3 dpi were identified by reference genome-guided transcriptome assembly (log_2_FC ≥ 1, FDR ≤ 0.05). File S1f. Differentially expressed genes (DEGs) in liver 10 dpi were identified by reference genome-guided transcriptome assembly (log_2_FC ≥ 1, FDR ≤ 0.05). File S2a. Differentially expressed transcripts (DETs) in head-kidney 3 dpi identified by reference genome-guided transcriptome assembly analysis (log_2_FC ≥ 1, FDR ≤ 0.05). File S2b. Differentially expressed transcripts (DETs) in head-kidney 10 days post-infection (dpi) were identified by reference genome-guided transcriptome assembly analysis (log_2_FC ≥ 1, FDR ≤ 0.05). File S2c. Differentially expressed transcripts (DETs) in spleen 3 dpi identified by reference genome-guided transcriptome assembly analysis (log_2_FC ≥ 1, FDR ≤ 0.05). File S2d. Differentially expressed transcripts (DETs) in spleen 10 dpi were identified by reference genome-guided transcriptome assembly analysis (log_2_FC ≥ 1, FDR ≤ 0.05). File S2e. Differentially expressed transcripts (DETs) in liver 3 dpi were identified by reference genome-guided transcriptome assembly analysis (log_2_FC ≥ 1, FDR ≤ 0.05). File S2f. Differentially expressed transcripts (DETs) in liver 10 dpi were identified by reference genome-guided transcriptome assembly analysis (log_2_FC ≥ 1, FDR ≤ 0.05). File S3a. Differentially expressed transcripts (DETs) in head-kidney 3 dpi identified by *de novo* transcriptome assembly analysis (log_2_FC ≥ 1, FDR ≤ 0.05). File S3b. Differentially expressed transcripts (DETs) in head-kidney 10 dpi identified by *de novo* transcriptome assembly analysis (log_2_FC ≥ 1, FDR ≤ 0.05). File S3c. Differentially expressed transcripts (DETs) in spleen 3 days post-infection (dpi) identified by *de novo* transcriptome assembly analysis (log_2_FC ≥ 1, FDR ≤ 0.05). File S3d. Differentially expressed transcripts (DETs) in spleen 10 dpi identified by *de novo* transcriptome assembly analysis (log_2_FC ≥ 1, FDR ≤ 0.05). File S3e. Differentially expressed transcripts (DETs) in liver 3 dpi identified by *de novo* transcriptome assembly analysis (log_2_FC ≥ 1, FDR ≤ 0.05). File S3f. Differentially expressed transcripts (DETs) in liver 10 dpi identified by *de novo* transcriptome assembly analysis (log_2_FC ≥ 1, FDR ≤ 0.05). File S4. nBlast results. File S5. List of all novel genes identified by *de novo* transcriptome analysis. File S6. Global view of gene ontology enrichment. File S7. Gene Ontology (GO) enrichment. File S8. Genes associated with the enriched pathway of interest. File S9a. Topmost significant genes in head-kidney 3 dpi. File S9b. Topmost significant genes in head-kidney 10 dpi. File S9c. Topmost significant genes in spleen 3 dpi. File S9d. Topmost significant genes in spleen 10 dpi. File S9e. Topmost significant genes in liver 3 dpi. File S9f. Topmost significant genes in liver 10 dpi. Suplementary Data 1: The data analyzed in this study are included in the supplement.

## References

[1] Eliasen K., Danielsen E., Johannesen Á., Joensen L.L., Patursson E.J. (2018). The cleaning efficacy of lumpfish (*Cyclopterus lumpus* L.) in Faroese salmon (*Salmo salar* L.) farming pens in relation to lumpfish size and seasonality. Aquaculture.

[2] Imsland A.K.D., Frogg N., Stefansson S.O., Reynolds P. (2019). Improving sea lice grazing of lumpfish (*Cyclopterus lumpus* L.) by feeding live feeds prior to transfer to Atlantic salmon (*Salmo salar* L.) net-pens. Aquaculture.

[3] Powell A., Treasurer J.W., Pooley C.L., Keay A.J., Lloyd R., Imsland A.K., Garcia de Leaniz C. (2018). Use of lumpfish for sea-lice control in salmon farming: Challenges and opportunities. Rev. Aquac..

[4] Brooker A.J., Papadopoulou A., Gutierrez C., Rey S., Davie A., Migaud H. (2018). Sustainable production and use of cleaner fish for the biological control of sea lice: Recent advances and current challenges. Vet. Reccord.

[5] Imsland A.K., Reynolds P., Eliassen G., Hangstad T.A., Foss A., Vikingstad E., Elvegård T.A. (2014). The use of lumpfish (*Cyclopterus lumpus* L.) to control sea lice (*Lepeophtheirus salmonis* Krøyer) infestations in intensively farmed Atlantic salmon (*Salmo salar* L.). Aquaculture.

[6] Imsland A.K.D., Hanssen A., Nytro A.V., Reynolds P., Jonassen T.M., Hangstad T.A., Elvegard T.A., Urskog T.C., Mikalsen B. (2018). It works! Lumpfish can significantly lower sea lice infestation in large-scale salmon farming. Biol. Open.

[7] Fish F.F. (1937). Furunculosis in Wild Trout. Copeia.

[8] Duijn J.C.V. (1962). Taxonomy of the Fish Furunculosis Organism. Nature.

[9] Janda J.M., Abbott S.L. (2010). The genus *Aeromonas*: Taxonomy, pathogenicity, and infection. Clininal Microbiol. Rev..

[10] Beaz-Hidalgo R., Figueras M.J. (2013). *Aeromonas* spp. whole genomes and virulence factors implicated in fish disease. J. Fish Dis..

[11] Gulla S., Duodu S., Nilsen A., Fossen I., Colquhoun D.J. (2016). *Aeromonas salmonicida* infection levels in pre- and post-stocked cleaner fish assessed by culture and an amended qPCR assay. J. Fish Dis..

[12] Rouleau F.D., Vincent A.T., Charette S.J. (2018). Genomic and phenotypic characterization of an atypical *Aeromonas salmonicida* strain isolated from a lumpfish and producing unusual granular structures. J. Fish Dis..

[13] Lin B., Chen S., Cao Z., Lin Y., Mo D., Zhang H., Gu J., Dong M., Liu Z., Xu A. (2007). Acute phase response in zebrafish upon *Aeromonas salmonicida* and *Staphylococcus aureus* infection: Striking similarities and obvious differences with mammals. Mol. Immunol..

[14] Long M., Nielsen T.K., Leisner J.J., Hansen L.H., Shen Z.X., Zhang Q.Q., Li A. (2016). *Aeromonas salmonicida* subsp. *salmonicida* strains isolated from Chinese freshwater fish contain a novel genomic island and possible regional-specific mobile genetic elements profiles. FEMS Microbiol. Lett..

[15] Ewart K.V., Belanger J.C., Williams J., Karakach T., Penny S., Tsoi S.C., Richards R.C., Douglas S.E. (2005). Identification of genes differentially expressed in Atlantic salmon (*Salmo salar*) in response to infection by *Aeromonas salmonicida* using cDNA microarray technology. Dev. Comp. Immunol..

[16] Haugland G.T., Jakobsen R.A., Vestvik N., Ulven K., Stokka L., Wergeland H.I. (2012). Phagocytosis and respiratory burst activity in lumpsucker (*Cyclopterus lumpus* L.) leucocytes analysed by flow cytometry. PLoS ONE.

[17] Ronneseth A., Ghebretnsae D.B., Wergeland H.I., Haugland G.T. (2015). Functional characterization of IgM+ B cells and adaptive immunity in lumpfish (*Cyclopterus lumpus* L.). Dev. Comp. Immunol..

[18] Patel D.M., Brinchmann M.F. (2017). Skin mucus proteins of lumpsucker (*Cyclopterus lumpus*). Biochem. Biophys. Rep..

[19] Eggestøl H.Ø., Lunde H.S., Rønneseth A., Fredman D., Petersen K., Mishra C.K., Furmanek T., Colquhoun D.J., Wergeland H.I., Haugland G.T. (2018). Transcriptome-wide mapping of signaling pathways and early immune responses in lumpfish leukocytes upon in vitro bacterial exposure. Sci. Rep..

[20] Gnanagobal H., Cao T., Hossain A., Dang M., Hall J.R., Kumar S., Van Cuong D., Boyce D., Santander J. (2021). Lumpfish (*Cyclopterus lumpus*) is susceptible to *Renibacterium salmoninarum* infection and induces cell-mediated immunity in the chronic stage. Front. Immunol..

[21] Ronneseth A., Haugland G.T., Colquhoun D.J., Brudal E., Wergeland H.I. (2017). Protection and antibody reactivity following vaccination of lumpfish (*Cyclopterus lumpus* L.) against atypical *Aeromonas salmonicida*. Fish. Shellfish Immunol..

[22] Erkinharju T., Lundberg M.R., Isdal E., Hordvik I., Dalmo R.A., Seternes T. (2017). Studies on the antibody response and side effects after intramuscular and intraperitoneal injection of Atlantic lumpfish (*Cyclopterus lumpus* L.) with different oil-based vaccines. J. Fish Dis..

[23] Dang M., Cao T., Vasquez I., Hossain A., Gnanagobal H., Kumar S., Hall J.R., Monk J., Boyce D., Westcott J. (2021). Oral immunization of larvae and juvenile of lumpfish (*Cyclopterus lumpus*) against *Vibrio anguillarum* does not influence systemic immunity. Vaccines.

[24] Zapata A., Diez B., Cejalvo T., Gutiérrez-de Frías C., Cortés A. (2006). Ontogeny of the immune system of fish. Fish. Shellfish Immunol..

[25] Tocher D.R. (2003). Metabolism and functions of lipids and fatty acids in teleost fish. Rev. Fish. Sci..

[26] Crispe I.N. (2009). The liver as a lymphoid organ. Annu. Rev. Immunol..

[27] Eckert C., Klein N., Kornek M., Lukacs-Kornek V. (2015). The complex myeloid network of the liver with diverse functional capacity at steady state and in inflammation. Front. Immunol..

[28] Thomson A.W., Knolle P.A. (2010). Antigen-presenting cell function in the tolerogenic liver environment. Nat. Rev. Immunol..

[29] González-Silvera D., Cuesta A., Esteban M.Á. (2021). Immune defence mechanisms presented in liver homogenates and bile of gilthead seabream (*Sparus aurata*). J. Fish. Biol..

[30] Frey J., Origgi F.C. (2016). Type III secretion system of *Aeromonas salmonicida* undermining the host’s immune response. Front. Mar. Sci..

[31] Valderrama K., Saravia M., Santander J. (2017). Phenotype of *Aeromonas salmonicida* sp. *salmonicida* cyclic adenosine 3’,5’-monophosphate receptor protein (Crp) mutants and its virulence in rainbow trout (*Oncorhynchus mykiss*). J. Fish Dis..

[32] Soto-Dávila M., Hossain A., Chakraborty S., Rise M.L., Santander J. (2019). *Aeromonas salmonicida* subsp. *salmonicida* early infection and immune response of Atlantic cod (*Gadus morhua* L.) primary macrophages. Front. Immunol..

[33] Soto-Dávila M., Valderrama K., Inkpen S.M., Hall J.R., Rise M.L., Santander J. (2020). Effects of vitamin D2 (Ergocalciferol) and D3 (Cholecalciferol) on Atlantic salmon (*Salmo salar*) primary macrophage immune response to *Aeromonas salmonicida* subsp. *salmonicida* infection. Front. Immunol..

[34] Vasquez I., Hossain A., Gnanagobal H., Valderrama K., Campbell B., Ness M., Charette S.J., Gamperl A.K., Cipriano R., Segovia C. (2022). Comparative Genomics of Typical and Atypical *Aeromonas salmonicida* Complete Genomes Revealed New Insights into Pathogenesis Evolution. Microorganisms.

[35] Leboffe M.J., Pierce B.E. (2015). Microbiology: Laboratory Theory & Application.

[36] Sambrook J., Russell W. (2001). Molecular Cloning, A Laboratory Manual.

[37] Chakraborty S., Woldemariam N.T., Visnovska T., Rise M.L., Boyce D., Santander J., Andreassen R. (2022). Characterization of miRNAs in Embryonic, Larval, and Adult Lumpfish Provides a Reference miRNAome for *Cyclopterus lumpus*. Biology.

[38] Vasquez I., Cao T., Hossain A., Valderrama K., Gnanagobal H., Dang M., Leeuwis R.H.J., Ness M., Campbell B., Gendron R. (2020). *Aeromonas salmonicida* infection kinetics and protective immune response to vaccination in sablefish (*Anoplopoma fimbria*). Fish. Shellfish Immunol..

[39] Reed L.J., Muench H. (1938). A simple method of estimating fifty per cent endpoints. Am. J. Hyg..

[40] Ahmed M. (2015). Acute Toxicity (Lethal Dose 50 Calculation) of Herbal Drug Somina in Rats and Mice. Pharmacol. Pharm..

[41] Chandler D., Roberson R.W. (2009). Bioimaging: Current Concepts in Light and Electron. Microscopy.

[42] Bibert S., Guex N., Lourenco J., Brahier T., Papadimitriou-Olivgeris M., Damonti L., Manuel O., Liechti R., Götz L., Tschopp J. (2021). Transcriptomic signature differences between SARS-CoV-2 and Influenza Virus infected patients. Front. Immunol..

[43] Jia Z., Wu N., Jiang X., Li H., Sun J., Shi M., Li C., Ge Y., Hu X., Ye W. (2021). Integrative transcriptomic analysis reveals the immune mechanism for a CyHV-3-Resistant common carp strain. Front. Immunol..

[44] Prokop J.W., Hartog N.L., Chesla D., Faber W., Love C.P., Karam R., Abualkheir N., Feldmann B., Teng L., McBride T. (2021). High-density blood transcriptomics reveals precision immune signatures of SARS-CoV-2 infection in hospitalized individuals. Front. Immunol..

[45] Roh H., Kim N., Lee Y., Park J., Kim B.S., Lee M.K., Park C.-I., Kim D.-H. (2021). Dual-organ transcriptomic analysis of rainbow trout infected with *Ichthyophthirius multifiliis* through co-expression and machine learning. Front. Immunol..

[46] Cai W., Kumar S., Navaneethaiyer U., Caballero-Solares A., Carvalho L.A., Whyte S.K., Purcell S.L., Gagne N., Hori T.S., Allen M. (2022). Transcriptome analysis of Atlantic salmon (*Salmo salar*) skin in response to sea lice and infectious Salmon Anemia Virus co-infection under different experimental functional diets. Front. Immunol..

[47] Cohen-Gihon I., Israeli O., Tidhar A., Sapoznikov A., Evgy Y., Stein D., Aftalion M., Gur D., Orr I., Zvi A. (2022). Transcriptome analysis of lungs in a mouse model of severe COVID-19. Front. Virol..

[48] Andrews S. (2010). FastQC: A Quality Control Tool for High Throughput Sequence Data.

[49] Ewels P., Magnusson M., Lundin S., Käller M. (2016). MultiQC: Summarize analysis results for multiple tools and samples in a single report. Bioinformatics.

[50] Li B., Ruotti V., Stewart R.M., Thomson J.A., Dewey C.N. (2009). RNA-Seq gene expression estimation with read mapping uncertainty. Bioinformatics.

[51] Teng M., Love M.I., Davis C.A., Djebali S., Dobin A., Graveley B.R., Li S., Mason C.E., Olson S., Pervouchine D. (2016). A benchmark for RNA-seq quantification pipelines. Genome Biol..

[52] Pereira M.B., Wallroth M., Jonsson V., Kristiansson E. (2018). Comparison of normalization methods for the analysis of metagenomic gene abundance data. BMC Genom..

[53] Robinson M.D., McCarthy D.J., Smyth G.K. (2009). edgeR: A Bioconductor package for differential expression analysis of digital gene expression data. Bioinformatics.

[54] Bolger A.M., Lohse M., Usadel B. (2014). Trimmomatic: A flexible trimmer for Illumina sequence data. Bioinformatics.

[55] Haas B.J., Papanicolaou A., Yassour M., Grabherr M., Blood P.D., Bowden J., Couger M.B., Eccles D., Li B., Lieber M. (2013). *De novo* transcript sequence reconstruction from RNA-seq using the Trinity platform for reference generation and analysis. Nat. Protoc..

[56] Li H., Handsaker B., Wysoker A., Fennell T., Ruan J., Homer N., Marth G., Abecasis G., Durbin R. (2009). Genome Project Data Processing, S., The sequence alignment/map format and SAMtools. Bioinformatics.

[57] Langmead B., Salzberg S.L. (2012). Fast gapped-read alignment with Bowtie 2. Nat. Methods.

[58] Robinson J.T., Thorvaldsdottir H., Winckler W., Guttman M., Lander E.S., Getz G., Mesirov J.P. (2011). Integrative genomics viewer. Nat. Biotechnol..

[59] Simao F.A., Waterhouse R.M., Ioannidis P., Kriventseva E.V., Zdobnov E.M. (2015). BUSCO: Assessing genome assembly and annotation completeness with single-copy orthologs. Bioinformatics.

[60] Li B., Dewey C.N. (2011). RSEM: Accurate transcript quantification from RNA-Seq data with or without a reference genome. BMC Bioinform..

[61] Bryant D.M., Johnson K., DiTommaso T., Tickle T., Couger M.B., Payzin-Dogru D., Lee T.J., Leigh N.D., Kuo T.-H., Davis F.G. (2017). A tissue-mapped axolotl *de novo* transcriptome enables identification of limb regeneration factors. Cell Rep..

[62] Finn R.D., Clements J., Eddy S.R. (2011). HMMER web server: Interactive sequence similarity searching. Nucleic Acids Res..

[63] Mistry J., Chuguransky S., Williams L., Qureshi M., Salazar G.A., Sonnhammer E.L.L., Tosatto S.C.E., Paladin L., Raj S., Richardson L.J. (2020). Pfam: The protein families database in 2021. Nucleic Acids Res..

[64] Almagro Armenteros J.J., Tsirigos K.D., Sønderby C.K., Petersen T.N., Winther O., Brunak S., von Heijne G., Nielsen H. (2019). SignalP 5.0 improves signal peptide predictions using deep neural networks. Nat. Biotechnol..

[65] Sonnhammer E.L., von Heijne G., Krogh A. (1998). A hidden Markov model for predicting transmembrane helices in protein sequences. Proc. Int. Conf. Intell. Syst. Mol. Biol..

[66] Love M.I., Huber W., Anders S. (2014). Moderated estimation of fold change and dispersion for RNA-seq data with DESeq2. Genome Biol..

[67] Bindea G., Mlecnik B., Hackl H., Charoentong P., Tosolini M., Kirilovsky A., Fridman W.H., Pagès F., Trajanoski Z., Galon J. (2009). ClueGO: A Cytoscape plug-in to decipher functionally grouped gene ontology and pathway annotation networks. Bioinformatics.

[68] Shannon P., Markiel A., Ozier O., Baliga N.S., Wang J.T., Ramage D., Amin N., Schwikowski B., Ideker T. (2003). Cytoscape: A software environment for integrated models of biomolecular interaction networks. Genome Res..

[69] St-Pierre J., Grégoire J.-C., Vaillancourt C. (2017). A simple method to assess group difference in RT-qPCR reference gene selection using GeNorm: The case of the placental sex. Sci. Rep..

[70] Soto-Dávila M., Chakraborty S., Santander J. (2022). Relative expression and validation of *Aeromonas salmonicida* subsp. *salmonicida* reference genes during ex vivo and in vivo fish infection. Infect. Genet. Evol..

[71] Roberts A., Schaeffer L., Pachter L. (2013). Updating RNA-Seq analyses after re-annotation. Bioinformatics.

[72] Steijger T., Abril J.F., Engström P.G., Kokocinski F., Abril J.F., Akerman M., Alioto T., Ambrosini G., Antonarakis S.E., Behr J. (2013). Assessment of transcript reconstruction methods for RNA-seq. Nat. Methods.

[73] Bjørgen H., Koppang E.O. (2021). Anatomy of teleost fish immune structures and organs. Immunogenetics.

[74] Zwollo P., Cole S., Bromage E., Kaattari S. (2005). B cell heterogeneity in the teleost kidney: Evidence for a maturation gradient from anterior to posterior kidney. J. Immunol..

[75] Flajnik M.F. (2018). A cold-blooded view of adaptive immunity. Nat. Rev. Immunol..

[76] Koppang E.O., Fischer U., Moore L., Tranulis M.A., Dijkstra J.M., Köllner B., Aune L., Jirillo E., Hordvik I. (2010). Salmonid T cells assemble in the thymus, spleen and in novel interbranchial lymphoid tissue. J. Anat..

[77] Lim J., Hong S. (2020). Characterization of *Aeromonas salmonicida* and *A. sobria* isolated from cultured salmonid fish in Korea and development of a vaccine against furunculosis. J. Fish Dis..

[78] Bricknell I.R., Bowden T.J., Bruno D.W., MacLachlan P., Johnstone R., Ellis A.E. (1999). Susceptibility of Atlantic halibut, *Hippoglossus hippoglossus* (L.) to infection with typical and atypical *Aeromonas salmonicida*. Aquaculture.

[79] Lin Q., Li J., Fu X., Liu L., Liang H., Niu Y., Huang C., Huang Z., Mo Z., Li N. (2020). Hemorrhagic gill disease of Chinese perch caused by *Aeromonas salmonicida* subsp. *salmonicida* in China. Aquaculture.

[80] Farto R., Milton D.L., Bermudez M.B., Nieto T.P. (2011). Colonization of turbot tissues by virulent and avirulent *Aeromonas salmonicida* subsp. *salmonicida* strains during infection. Dis. Aquat. Org..

[81] Braden L.M., Whyte S.K., Brown A.B.J., Iderstine C.V., Letendre C., Groman D., Lewis J., Purcell S.L., Hori T., Fast M.D. (2019). Vaccine-induced protection against furunculosis involves pre-emptive priming of humoral immunity in Arctic Charr. Front. Immunol..

[82] Turner K.H., Everett J., Trivedi U., Rumbaugh K.P., Whiteley M. (2014). Requirements for *Pseudomonas aeruginosa* acute burn and chronic surgical wound infection. PLoS Genet..

[83] Wu Z., Wang X., Zhang X. (2011). Using non-uniform read distribution models to improve isoform expression inference in RNA-Seq. Bioinformatics.

[84] Mortazavi A., Williams B.A., McCue K., Schaeffer L., Wold B. (2008). Mapping and quantifying mammalian transcriptomes by RNA-Seq. Nat. Methods.

[85] Glaus P., Honkela A., Rattray M. (2012). Identifying differentially expressed transcripts from RNA-seq data with biological variation. Bioinformatics.

[86] Cloonan N., Forrest A.R., Kolle G., Gardiner B., Faulkner G.J., Brown M.K., Taylor D.F., Steptoe A.L., Wani S., Bethel G. (2008). Stem cell transcriptome profiling via massive-scale mRNA sequencing. Nat. Methods.

[87] Marioni J.C., Mason C.E., Mane S.M., Stephens M., Gilad Y. (2008). RNA-seq: An assessment of technical reproducibility and comparison with gene expression arrays. Genome Res..

[88] Wang X., Wu Z., Zhang X. (2010). Isoform abundance inference provides a more accurate estimation of gene expression levels in RNA-seq. J. Bioinform. Comput. Biol..

[89] Yi L., Pimentel H., Bray N.L., Pachter L. (2018). Gene-level differential analysis at transcript-level resolution. Genome Biol..

[90] Martin J.A., Wang Z. (2011). Next-generation transcriptome assembly. Nat. Rev. Genet..

[91] Kovi M.R., Abdelhalim M., Kunapareddy A., Ergon Å., Tronsmo A.M., Brurberg M.B., Hofgaard I.S., Asp T., Rognli O.A. (2016). Global transcriptome changes in perennial ryegrass during early infection by pink snow mould. Sci. Rep..

[92] Grabherr M.G., Haas B.J., Yassour M., Levin J.Z., Thompson D.A., Amit I., Adiconis X., Fan L., Raychowdhury R., Zeng Q. (2011). Full-length transcriptome assembly from RNA-Seq data without a reference genome. Nat. Biotechnol..

[93] Langmead B., Trapnell C., Pop M., Salzberg S.L. (2009). Ultrafast and memory-efficient alignment of short DNA sequences to the human genome. Genome Biol..

[94] Trapnell C., Williams B.A., Pertea G., Mortazavi A., Kwan G., Van Baren M.J., Salzberg S.L., Wold B.J., Pachter L. (2010). Transcript assembly and quantification by RNA-Seq reveals unannotated transcripts and isoform switching during cell differentiation. Nat. Biotechnol..

[95] Ward J.A., Ponnala L., Weber C.A. (2012). Strategies for transcriptome analysis in non-model plants. Am. J. Bot..

[96] Lee S.G., Na D., Park C. (2021). Comparability of reference-based and reference-free transcriptome analysis approaches at the gene expression level. BMC Bioinform..

[97] Kovi M.R., Amdahl H., Alsheikh M., Rognli O.A. (2017). De novo and reference transcriptome assembly of transcripts expressed during flowering provide insight into seed setting in tetraploid red clover. Sci. Rep..

[98] Lu B., Zeng Z., Shi T. (2013). Comparative study of *de novo* assembly and genome-guided assembly strategies for transcriptome reconstruction based on RNA-Seq. Sci. China. Life Sci..

[99] Vijay N., Poelstra J.W., Künstner A., Wolf J.B. (2013). Challenges and strategies in transcriptome assembly and differential gene expression quantification. A comprehensive in silico assessment of RNA-seq experiments. Mol. Ecol..

[100] Kumar R., Joy K.P., Singh S.M. (2016). Morpho-histology of head kidney of female catfish *Heteropneustes fossilis*: Seasonal variations in melano-macrophage centers, melanin contents and effects of lipopolysaccharide and dexamethasone on melanins. Fish. Physiol. Biochem..

[101] Morales-Lange B., Agboola J.O., Hansen J.Ø., Lagos L., Øyås O., Mercado L., Mydland L.T., Øverland M. (2021). The Spleen as a Target to Characterize Immunomodulatory Effects of Down-Stream Processed *Cyberlindnera jadinii* Yeasts in Atlantic salmon exposed to a dietary soybean meal challenge. Front. Immunol..

[102] Gong H., Wang Q., Lai Y., Zhao C., Sun C., Chen Z., Tao J., Huang Z. (2021). Study on immune response of organs of Epinephelus coioides and Carassius auratus after immersion vaccination with inactivated *Vibrio harveyi* vaccine. Front. Immunol..

[103] Bronte V., Pittet M.J. (2013). The spleen in local and systemic regulation of immunity. Immunity.

[104] Rui L. (2014). Energy metabolism in the liver. Comp. Physiol..

[105] Charlie-Silva I., Klein A., Gomes J.M.M., Prado E.J.R., Moraes A.C., Eto S.F., Fernandes D.C., Fagliari J.J., Junior J.D.C., Lima C. (2019). Acute-phase proteins during inflammatory reaction by bacterial infection: Fish-model. Sci. Rep..

[106] Uhlar C.M., Whitehead A.S. (1999). Serum amyloid A, the major vertebrate acute-phase reactant. Eur. J. Biochem..

[107] Dinarello C.A. (2018). Overview of the IL-1 family in innate inflammation and acquired immunity. Immunol. Rev..

[108] Rebl A., Korytar T., Kobis J.M., Verleih M., Krasnov A., Jaros J., Kuhn C., Kollner B., Goldammer T. (2014). Transcriptome profiling reveals insight into distinct immune responses to *Aeromonas salmonicida* in gill of two rainbow trout strains. Mar. Biotechnol..

[109] Long M., Zhao J., Li T., Tafalla C., Zhang Q., Wang X., Gong X., Shen Z., Li A. (2015). Transcriptomic and proteomic analyses of splenic immune mechanisms of rainbow trout (*Oncorhynchus mykiss*) infected by *Aeromonas salmonicida* subsp. *salmonicida*. J. Proteom..

[110] Stearns-Kurosawa D.J., Osuchowski M.F., Valentine C., Kurosawa S., Remick D.G. (2011). The pathogenesis of sepsis. Annu. Rev. Pathol..

[111] O’Brien J.M., Ali N.A., Aberegg S.K., Abraham E. (2007). Sepsis. Am. J. Med..

[112] Smith N.C., Rise M.L., Christian S.L. (2019). A Comparison of the innate and adaptive immune systems in cartilaginous fish, ray-finned fish, and lobe-finned fish. Front. Immunol..

[113] Cohen J. (2002). The immunopathogenesis of sepsis. Nature.

[114] Amara U., Flierl M.A., Rittirsch D., Klos A., Chen H., Acker B., Brückner U.B., Nilsson B., Gebhard F., Lambris J.D. (2010). Molecular intercommunication between the complement and coagulation systems. J. Immunol..

[115] Antoniak S. (2018). The coagulation system in host defense. Res. Pract. Thromb. Haemost..

[116] Semeraro N., Ammollo C.T., Semeraro F., Colucci M. (2010). Sepsis-associated disseminated intravascular coagulation and thromboembolic disease. Mediterr. J. Hematol. Infect. Dis..

[117] Levi M., van der Poll T. (2017). Coagulation and sepsis. Thromb. Res..

[118] Goeijenbier M., van Wissen M., van de Weg C., Jong E., Gerdes V.E.A., Meijers J.C.M., Brandjes D.P.M., van Gorp E.C.M. (2012). Review: Viral infections and mechanisms of thrombosis and bleeding. J. Med. Virol..

[119] Lupu F., Keshari R.S., Lambris J.D., Mark Coggeshall K. (2014). Crosstalk between the coagulation and complement systems in sepsis. Thromb. Res..

[120] Pierrakos C., Vincent J.-L. (2010). Sepsis biomarkers: A review. Crit. Care.

[121] Fast M.D., Tse B., Boyd J.M., Johnson S.C. (2009). Mutations in the Aeromonas salmonicida subsp. *salmonicida* type III secretion system affect Atlantic salmon leucocyte activation and downstream immune responses. Fish. Shellfish Immunol..

[122] Vanden Bergh P., Frey J. (2014). *Aeromonas salmonicida* subsp. *salmonicida* in the light of its type-three secretion system. Microb. Biotechnol..

[123] Vanden Bergh P., Heller M., Braga-Lagache S., Frey J. (2013). The *Aeromonas salmonicida* subsp. *salmonicida* exoproteome: Global analysis, moonlighting proteins and putative antigens for vaccination against furunculosis. Proteome Sci..

[124] Vanden Bergh P., Heller M., Braga-Lagache S., Frey J. (2013). The *Aeromonas salmonicida* subsp. *salmonicida* exoproteome: Determination of the complete repertoire of Type-Three Secretion System effectors and identification of other virulence factors. Proteome Sci..

[125] Radaev S., Zou Z., Tolar P., Nguyen K., Nguyen A., Krueger P.D., Stutzman N., Pierce S., Sun P.D. (2010). Structural and functional studies of Ig alpha beta and its assembly with the B cell antigen receptor. Structure.

[126] Jiang S., Sun L. (2017). Tongue Sole CD209: A Pattern-Recognition Receptor that Binds a Broad Range of Microbes and Promotes Phagocytosis. Int. J. Mol. Sci..

[127] Dallaire-Dufresne S., Tanaka K.H., Trudel M.V., Lafaille A., Charette S.J. (2014). Virulence, genomic features, and plasticity of *Aeromonas salmonicida* subsp. salmonicida, the causative agent of fish furunculosis. Vet. Microbiol..

[128] Machado A.M., Figueiredo C., Seruca R., Rasmussen L.J. (2010). Helicobacter pylori infection generates genetic instability in gastric cells. Biochim. Biophys. Acta.

[129] Rich T., Allen R.L., Wyllie A.H. (2000). Defying death after DNA damage. Nature.

[130] Zgur-Bertok D. (2013). DNA damage repair and bacterial pathogens. PLoS Pathog..

[131] Sun B., van Dissel D., Mo I., Boysen P., Haslene-Hox H., Lund H. (2022). Identification of novel biomarkers of inflammation in Atlantic salmon (*Salmo salar* L.) by a plasma proteomic approach. Dev. Comp. Immunol.

[132] Barichello T., Generoso J.S., Singer M., Dal-Pizzol F. (2022). Biomarkers for sepsis: More than just fever and leukocytosis—a narrative review. Crit. Care.

[133] Schrödl W., Büchler R., Wendler S., Reinhold P., Muckova P., Reindl J., Rhode H. (2016). Acute phase proteins as promising biomarkers: Perspectives and limitations for human and veterinary medicine. Proteomics. Clin. Appl..

