# Supplementary material for: Multi-Organ Transcriptome Response of Lumpfish (Cyclopterus lumpus) to Aeromonas salmonicida Subspecies salmonicida Systemic Infection
Source: Microorganisms. 2022 Oct 26;10(11):2113. doi: 10.3390/microorganisms10112113 (PMC9692985; doi:10.3390/microorganisms10112113)
Supplement: Supplementary file 1 [file microorganisms-10-02113-s001.zip › microorganisms-1974933-supplementary2/microorganisms-1974933-supplementary/Supporting information/Presentation 1.pptx]

## Slide 1
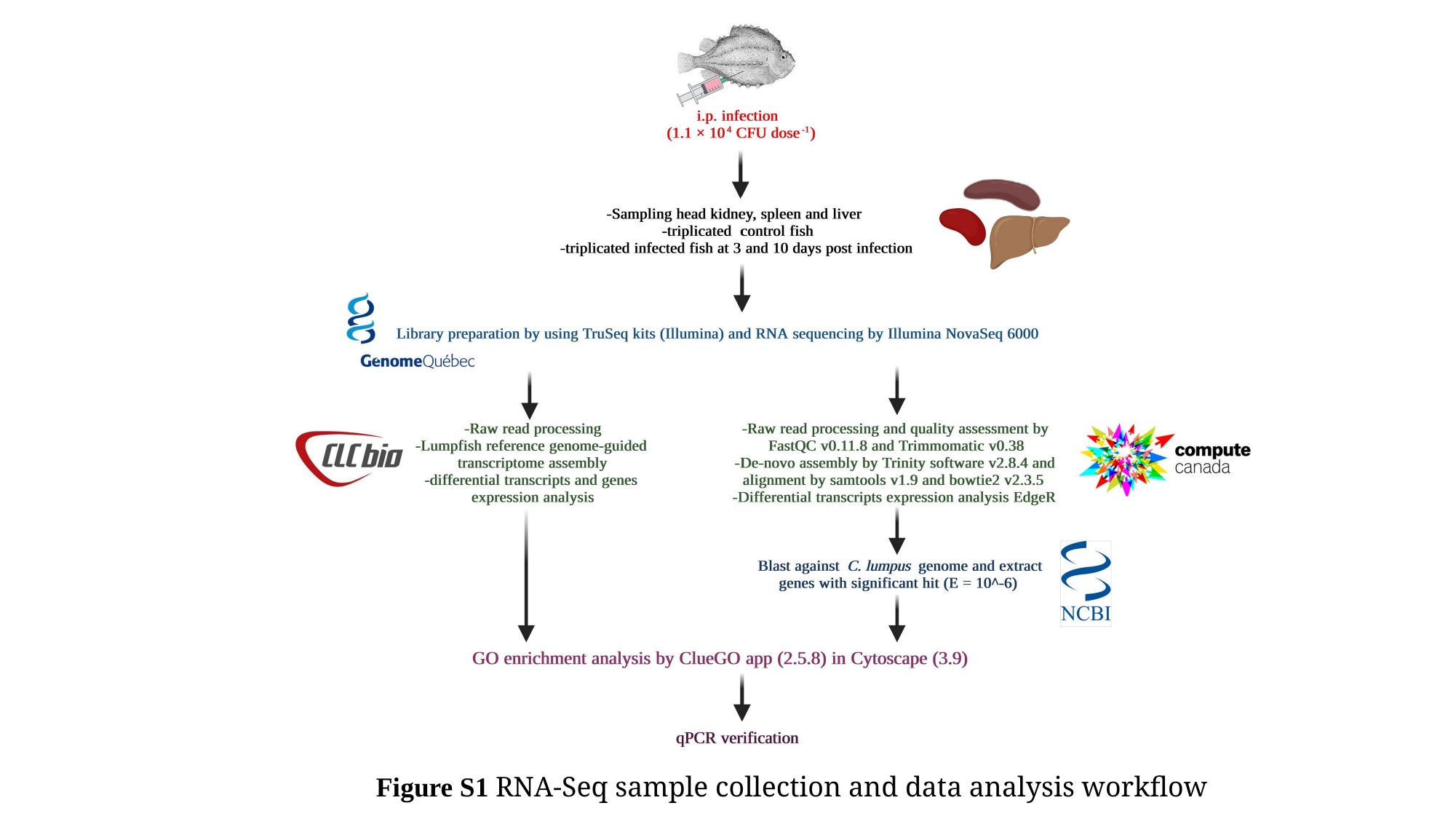

Figure S1 RNA-Seq sample collection and data analysis workflow

## Slide 2
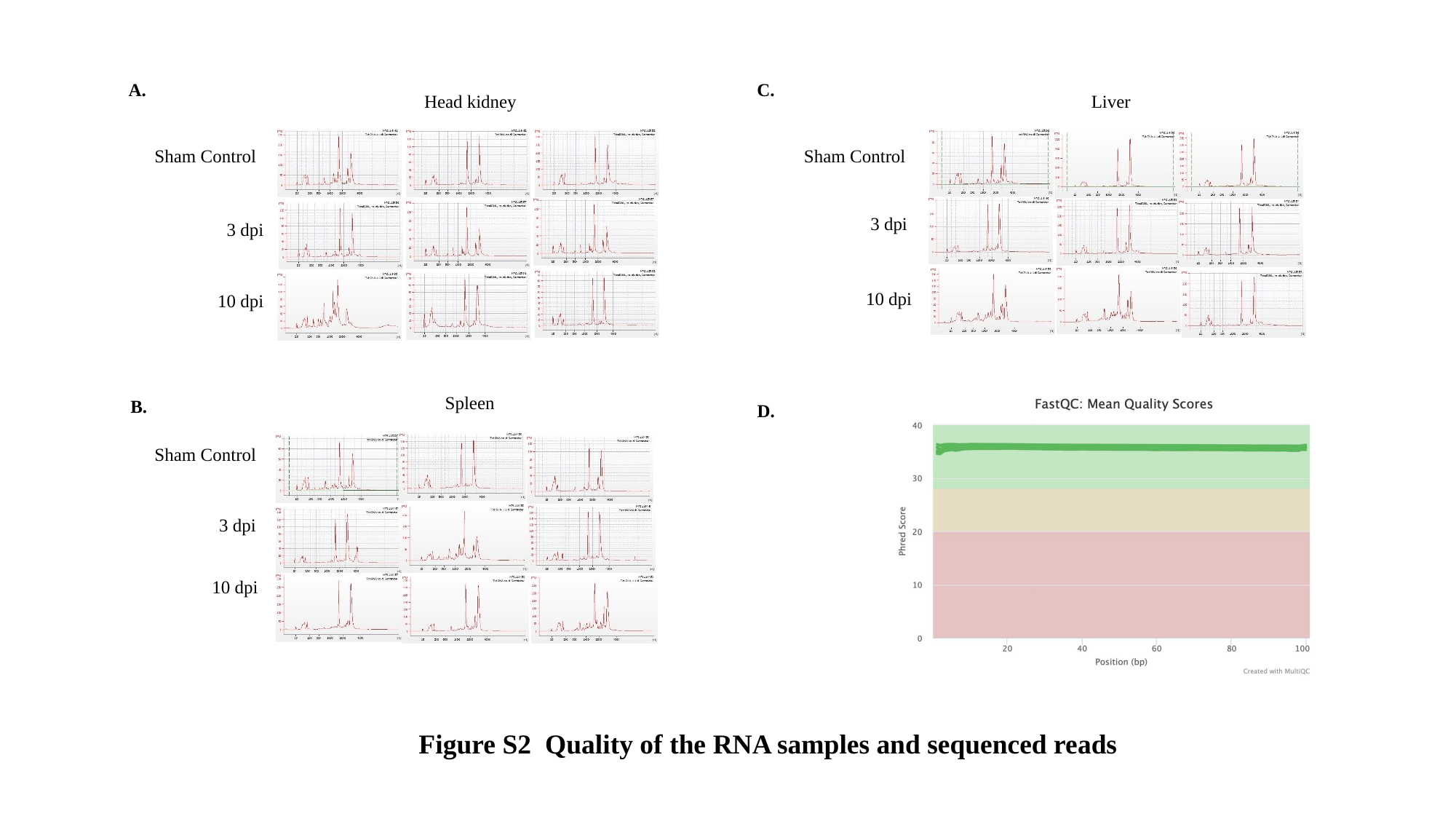

A.
 C.
Head kidney
Liver
Sham Control
Sham Control
3 dpi
3 dpi
10 dpi
10 dpi
Spleen
 B.
 D.
Sham Control
3 dpi
10 dpi
Figure S2 Quality of the RNA samples and sequenced reads

## Slide 3
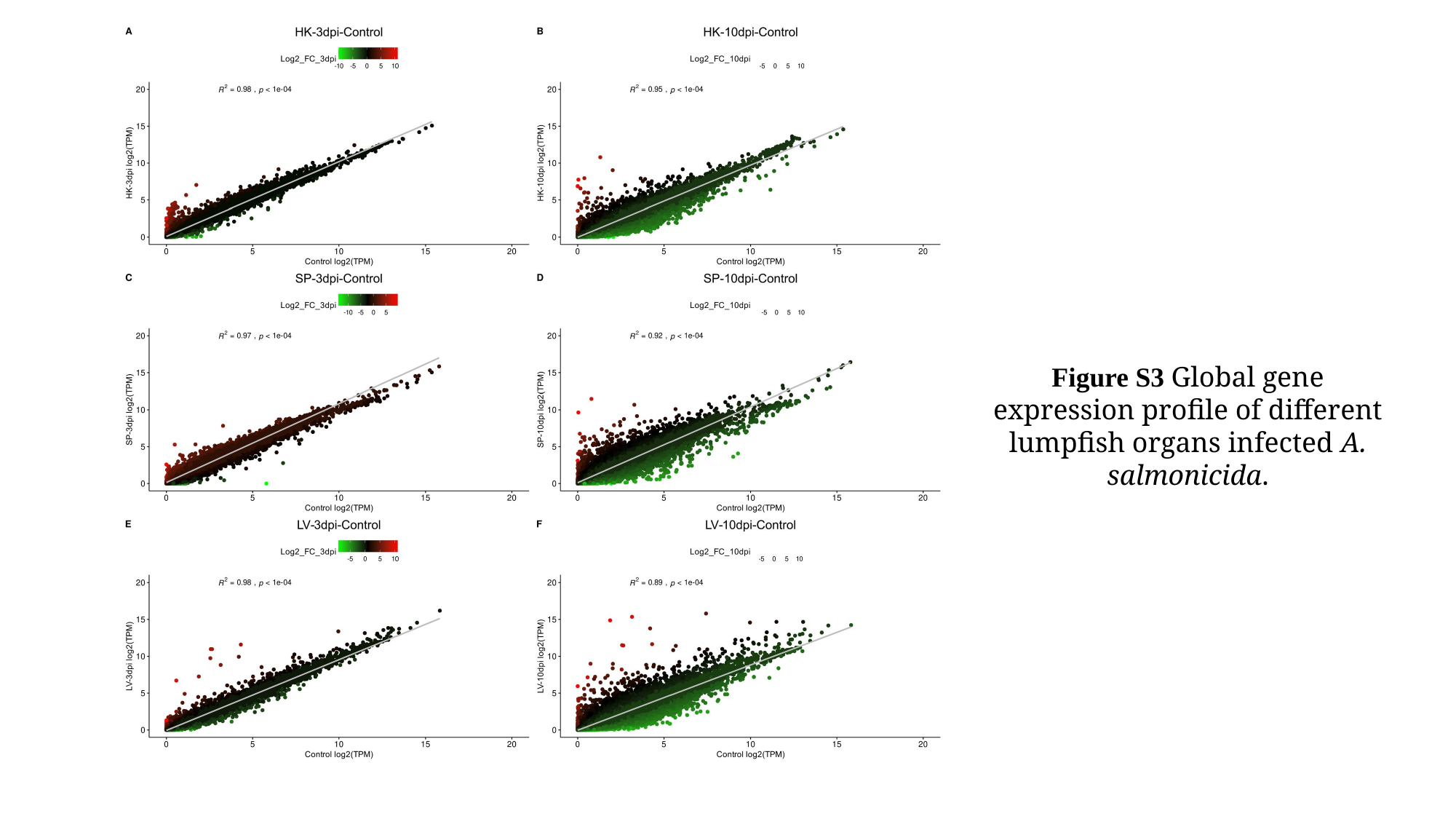

Figure S3 Global gene expression profile of different lumpfish organs infected A. salmonicida.

## Slide 4
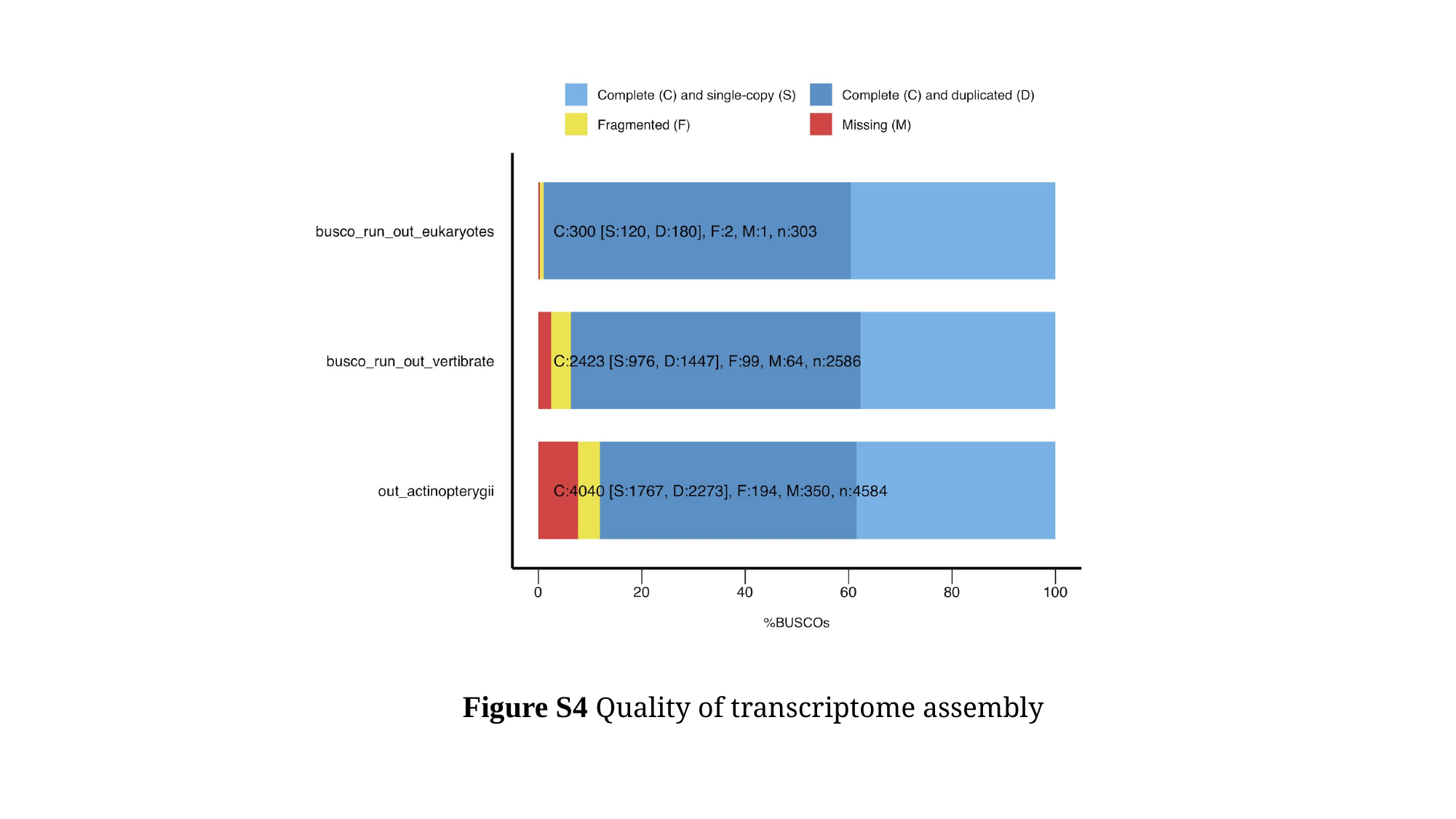

Figure S4 Quality of transcriptome assembly

## Slide 5
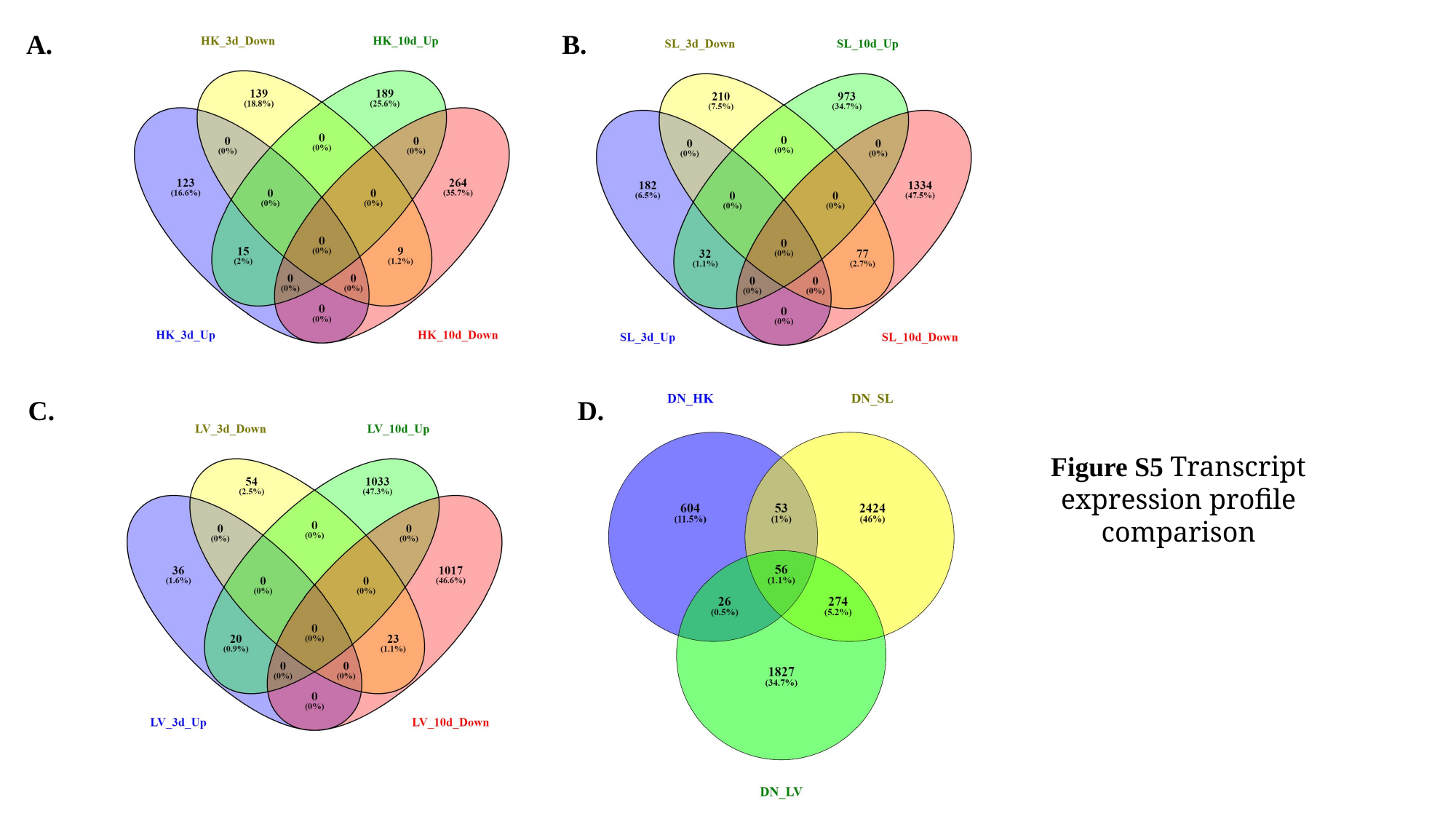

A.
B.
D.
C.
Figure S5 Transcript expression profile comparison

## Slide 6
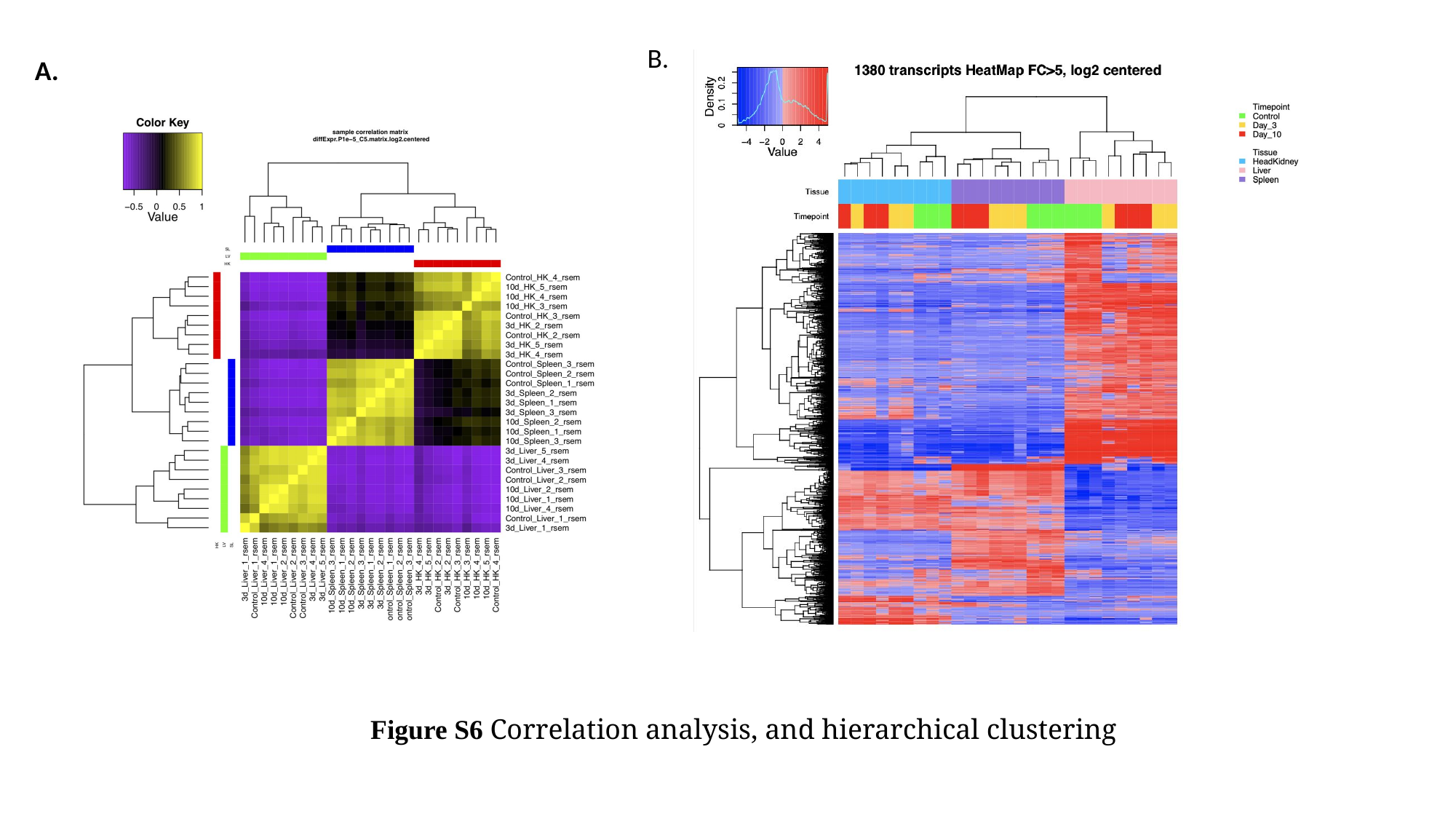

B.
A.
Figure S6 Correlation analysis, and hierarchical clustering

## Slide 7
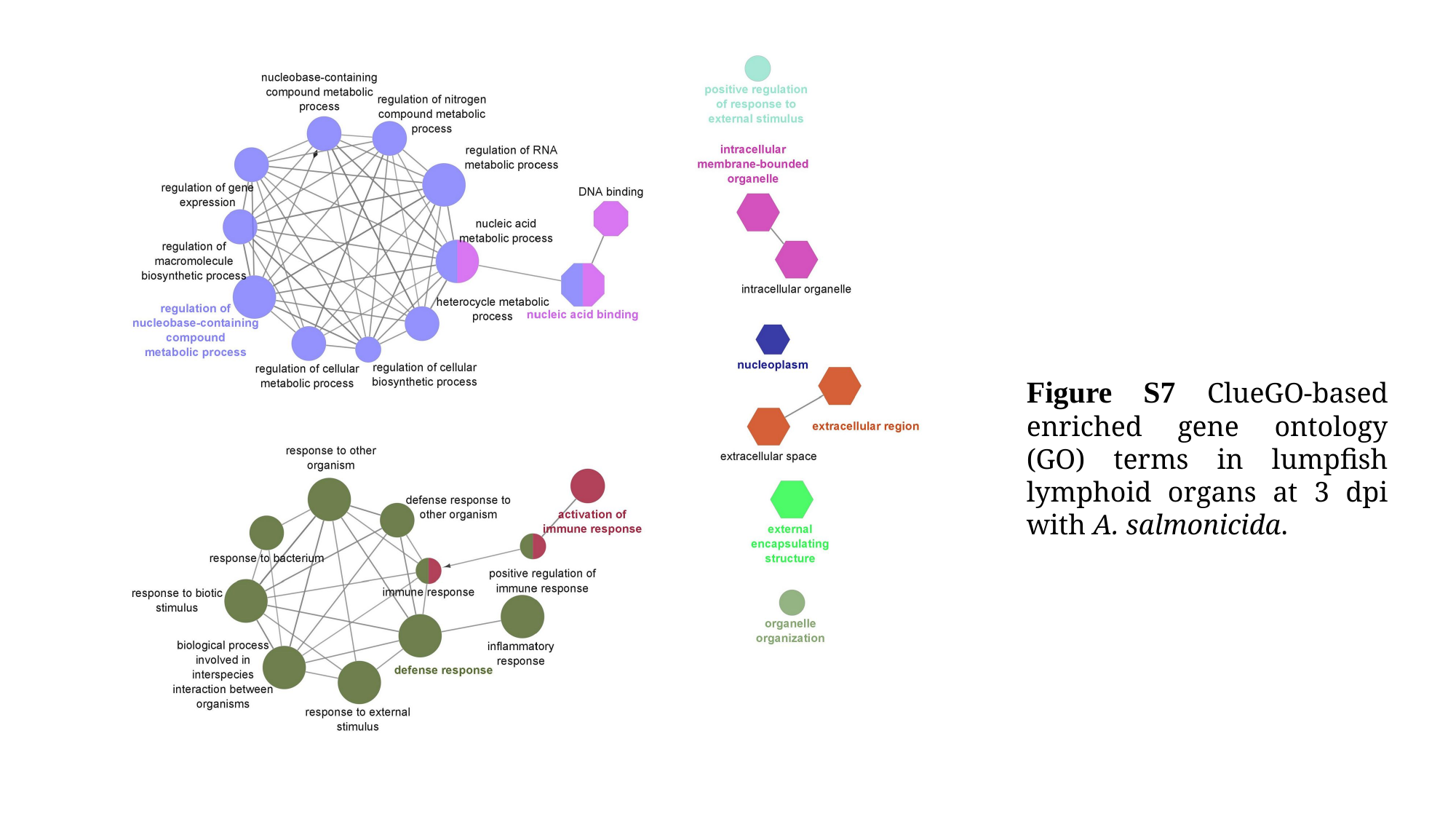

Figure S7 ClueGO-based enriched gene ontology (GO) terms in lumpfish lymphoid organs at 3 dpi with A. salmonicida.

## Slide 8
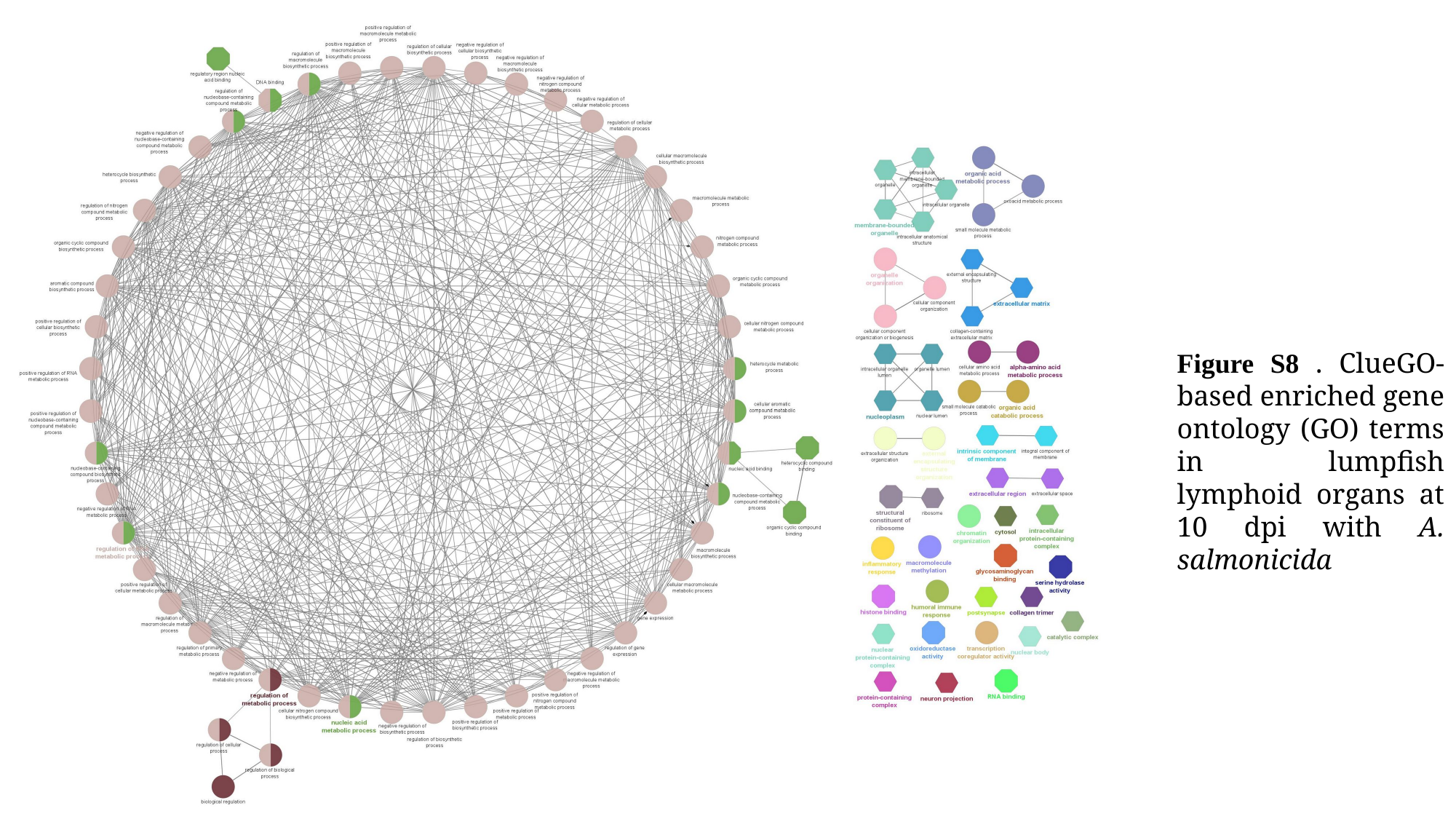

Figure S8 . ClueGO-based enriched gene ontology (GO) terms in lumpfish lymphoid organs at 10 dpi with A. salmonicida

## Slide 9
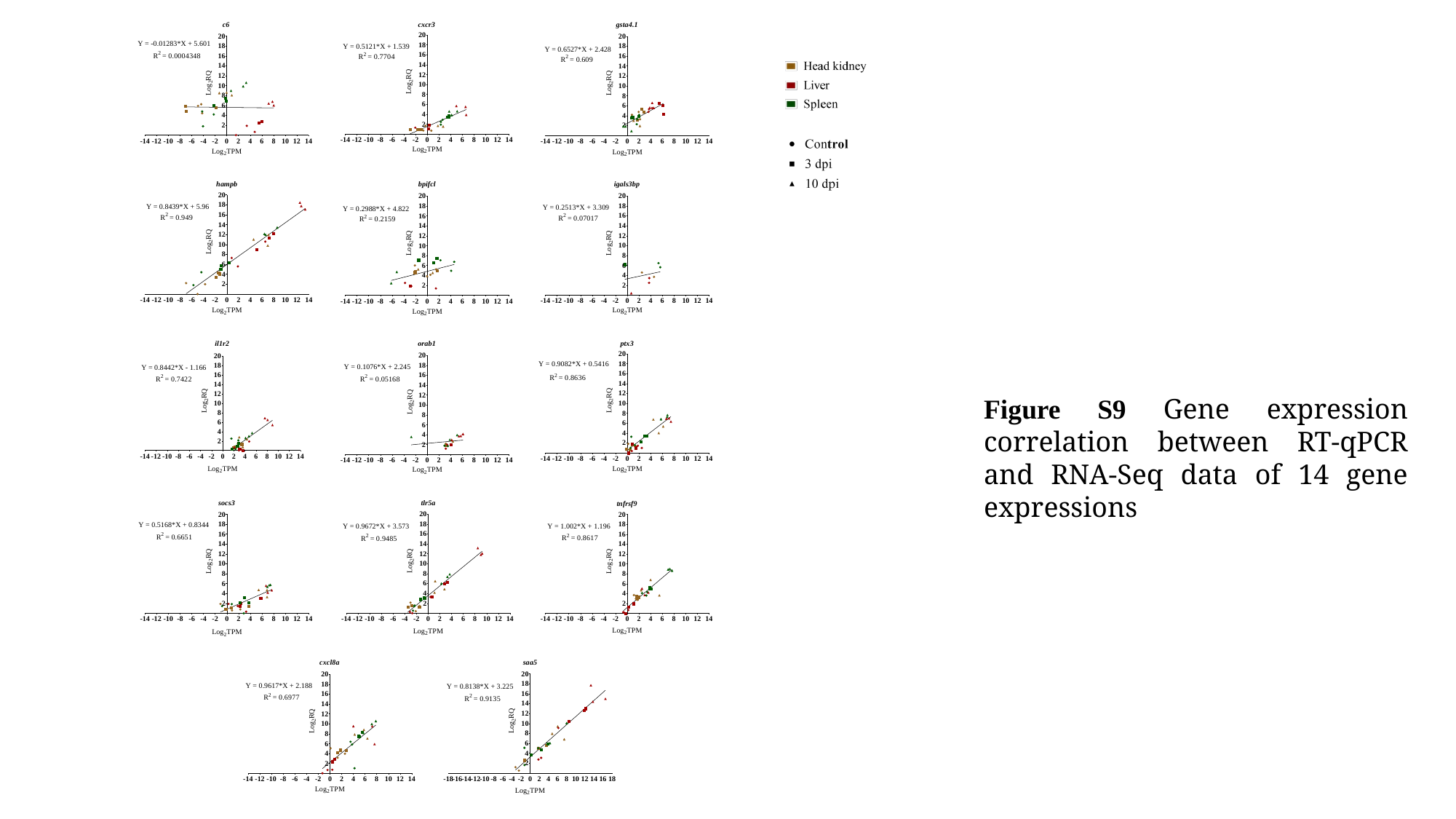

Figure S9 Gene expression correlation between RT-qPCR and RNA-Seq data of 14 gene expressions

## Slide 10
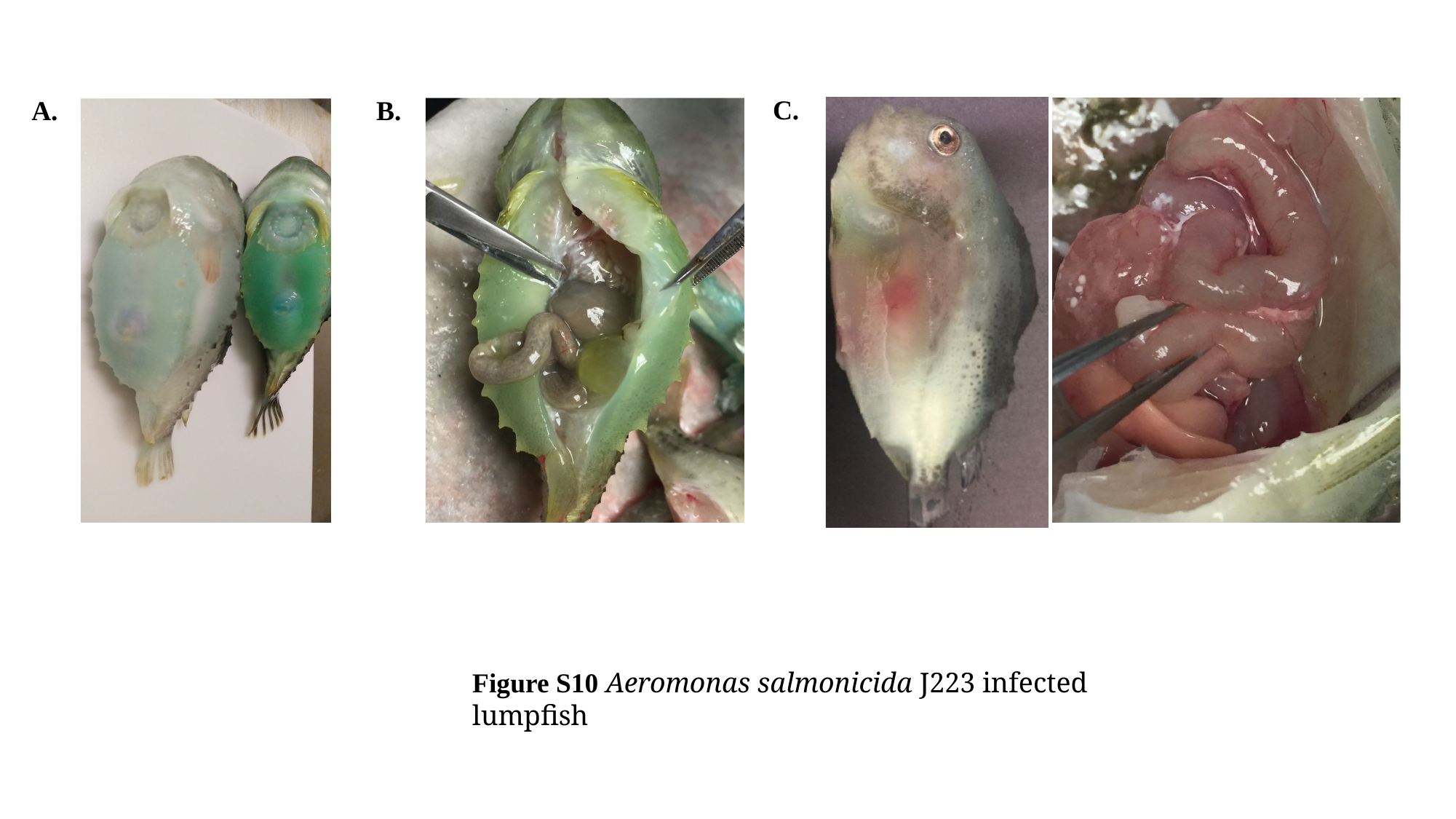

C.
A.
B.
Figure S10 Aeromonas salmonicida J223 infected lumpfish
