# Supplementary material for: Multi-Organ Transcriptome Response of Lumpfish (Cyclopterus lumpus) to Aeromonas salmonicida Subspecies salmonicida Systemic Infection
Source: Microorganisms. 2022 Oct 26;10(11):2113. doi: 10.3390/microorganisms10112113 (PMC9692985; doi:10.3390/microorganisms10112113)
Supplement: Supplementary file 1 [file microorganisms-10-02113-s001.zip › microorganisms-1974933-supplementary2/microorganisms-1974933-supplementary/Supporting information/Supplementary tables.pdf]

Table S1. Quality of the RNA

Sample name	28s/18s	RIN	Concentration (ng/µl)	260/230	260/280
Control _Head kidney_1	1.53	9.2	852.84	2.31	2.07
Control _ Head kidney _2	0.90	7.8	955.81	2.19	2.06
Control _ Head kidney _3	1.48	9.3	299.9	2.24	2.05
3 dpi_ Head kidney _1	1.12	8.7	280.09	1.29	2.07
3 dpi_ Head kidney _2	0.65	6.8	847.86	2.1	2.09
3 dpi_ Head kidney _3	0.91	8.2	227.22	1.67	2.08
10 dpi_ Head kidney _1	1.11	8.7	229.25	1.79	2.02
10 dpi_ Head kidney _2	1.36	9	225.24	1.51	2.06
10 dpi_ Head kidney _3	0.50	5.4	1083.98	2.33	2.00
Control_Spleen_1	0.94	7.6	356.49	1.52	2.03
Control_Spleen _2	1.66	8.6	366.55	2.16	2.05
Control_Spleen _3	1.08	8.7	286.33	2.05	2.05
3 dpi_ Spleen _1	1.42	9.2	521.28	1.98	2.08
3 dpi_ Spleen _2	1.67	9.5	339.04	1.56	2.04
3 dpi_ Spleen _3	1.62	9	470.4	2.33	1.95
10 dpi_ Spleen _1	1.01	6.8	875.61	2.31	2.05
10 dpi_ Spleen _2	0.81	6.7	687.89	2.27	2.05
10 dpi_ Spleen _3	1.30	8.7	639.58	1.91	2.06
Control_Liver_1	1.18	8.6	346.84	1.52	2.03
Control_Liver _2	1.7	7.6	356.49	2.16	2.05
Control_Liver _3	1.32	10	912.41	2.05	2.05
3 dpi_ Liver _1	0.56	7.3	927.85	1.46	2.08
3 dpi_ Liver _2	1.62	9.6	586.1	1.85	2.09
3 dpi_ Liver _3	1.65	9.5	466.16	2.09	1.99
10 dpi_ Liver _1	1.13	8.7	780.28	2.23	2.06
10 dpi_ Liver _2	1.04	7.4	1351.75	2.33	2.05
10 dpi_ Liver _3	1.81	9.8	522.96	1.96	2.08

Table S2. Primers used in this study

Gene name (*Gene symbol*) (Trinity ID)	Nucleotide sequence (5'-3')	ʻEfficiency (%)	Amplicon size (bp)
complement component 6 (*c6*) (DN137_c0_g1_i25)	F: CTGTCACCCCTCCACAGAGT	98.8	100
	R: GTTGCATGTCAGCGTTGAGT		
C-X-C motif chemokine receptor 3 (LOC117745182 /*cxcr3*)	F: AGAGTTCACTGTGGGGGTTG	93.9	106
(DN2498_c0_g1_i8)	R: GACTGCACCTGGTGACCTTT		
galectin-3-binding protein (LOC117735282/ *igals3bp*) (DN1760_c1_g1_i3)	F: GTGCCTCAGAACGGTCTCTC	96	114
	R: GGCCATGTTGTCCTTGAAGT		
glutathione S-transferase alpha 4 (*gsta4.1*)	F: CCTATGGTGGAAATGGATGG	92.9	108
(DN1192_c0_g1_i2)	R: CATGACCCGGTCTTTGAGAT		
hepcidin anti-microbial peptide b (LOC117728128 / *hampb*) (DN2492_c0_g1_i7)	F: CGTCGTGCTCACCTTCATTT	91.9	95
	R: CTGGGTTGTCAACGCTCAT		
interleukin 1 receptor type 2 (LOC117750249/ *il1r2*) (DN49226_c0_g1_i2)	F: CTCATTGATGAGCGGCAGTA	92.5	111
	R: GGGGTCAGAGGTCACAGAGA		
interleukin 8b (*il8b*) alias C-X-C motif chemokine ligand 8b (*cxcl8b*)	F: GTCTGAGAAGCCTGGGAGTG	87.4	138
(DN2906_c0_g1_i1)	R: TCAGAGTGGCAATGATCTCG		

BPI fold-containing family C- like (*bpifcl*) (DN1675_c0_g2_i3)	F: GTTTCCCGGACTTCTGATGA<br>R: GGTACCATTTGGTTGGATGG	95.7	130
pentraxin 3 (*ptx3a*)<br>(DN6917_c2_g1_i1)	F: GCCTCAAACCCAGAGATCAG<br>R: GTCGGGAAGTTTGCATTTGT	90	105
ras-related protein ORAB-1 (LOC117743939/ *orab1*) (DN1973_c0_g3_i2)	F: ACAGGAGATCGACCGCTATG<br>R: GAGTCTGCGAACTCCTTTGC	92.4	117
serum amyloid A 5 (LOC117728776/ *saa5*) (DN17527_c0_g1_i1)	F: AGAGTGGGTGCAGGAAAGAA<br>R: GAAGTCCTGGTGGCCTGTAA	92.1	116
suppressor of cytokine signaling 3 (*socs3a*) (DN488_c0_g1_i2)	F: CATGCCTCAGAGCAAAGTGA<br>R: AGCTGCAGGAGAGAGGTCTG	93.7	104
TNF receptor superfamily member 9 (LOC117733297/ *tnfrsf9*) (DN2095_c0_g3_i4)	F: AGGAGAAGAAAAGCCGATCC<br>R: CTCGTGGAAACTGCACTCAA	94.4	115
toll-like receptor 5a (LOC117727165/ *tlr5a*) (DN8688_c0_g1_i1)	F: TGGACGAGTTTCAGCAGTTG	92.9	129

Table S3. Trimming and mapping statistics.

Experimental conditions	Number of reads before trim	Number of reads after trim	Percentage trimmed (%)	Number of mapped reads	% of mapped reads
HK1_control	90,833,894	89,460,642	98.49	88,772,934	99.23127
HK2_control	82,357,896	81,214,335	98.61	80,651,238	99.30665
HK3_control	95,840,032	94,471,719	98.57	93,794,340	99.28298
HK1_3 dpi	85,364,678	84,249,691	98.69	83,689,892	99.33555
HK2_3 dpi	69,166,462	68,321,181	98.78	67,915,962	99.40689
HK3_3 dpi	83,210,266	82,006,542	98.55	81,417,744	99.28201
HK1_10 dpi	74,687,068	73,641,866	98.6	80,640,940	109.5042
HK2_10 dpi	75,696,762	74,572,712	98.52	73,123,686	98.0569
HK3_10 dpi	83,134,430	81,500,846	98.04	74,020,234	90.82143
LV1_control	75,238,962	74,186,108	98.6	73,667,016	99.30028
LV2_control	82,613,962	81,592,390	98.76	81,097,032	99.39289
LV3_control	89,149,382	87,873,344	98.57	87,249,434	99.28999
LV1_3 dpi	73,482,382	72,624,442	98.83	72,205,844	99.42361
LV2_3 dpi	76,781,706	75,902,586	98.86	75,475,798	99.43772
LV3_3 dpi	78,365,294	77,336,892	98.69	76,810,090	99.31882
LV1_10 dpi	66,590,420	65,684,895	98.64	73,681,162	112.1737
LV2_10 dpi	84,053,066	82,945,239	98.68	72,873,446	87.8573
LV3_10 dpi	72,837,270	71,790,152	98.56	69,948,408	97.43455
SL1_control	82,894,516	81,638,490	98.48	81,023,884	99.24716
SL2_control	85,830,500	84,711,276	98.7	84,168,702	99.3595
SL3_control	80,510,892	79,352,845	98.56	78,781,698	99.28024
SL1_3 dpi	67,505,126	66,241,078	98.13	65,565,504	98.98013
SL2_3 dpi	95,481,322	94,028,713	98.48	93,300,368	99.2254
SL3_3 dpi	79,665,640	78,523,117	98.57	77,967,908	99.29294
SL1_10 dpi	75,150,950	74,159,068	98.68	65,230,402	87.96012
SL2_10 dpi	74,330,220	73,364,626	98.7	82,383,274	112.2929

SL3_10 dpi	71,265,916	70,388,130	98.77	71,286,900	101.2769

Table S4. Trinity statistics

Total trinity transcripts	403204
Total trinity genes	270150
Percent GC	45.99
Contig N10	9165
Contig N20	6690
Contig N30	5231
Contig N40	4164
Contig N50	3235
Median contig length	497
Average contig	1296.15
Total assembled bases	522614427

Table S5. Alignment statistics

**Sample name**	**Aligned concordantly exactly 1 time**	**Aligned concordantly >1 times**	**Overall alignment rate (%)**
Control _Head kidney_1	6056815 (14%)	33309331 (76.98%)	97.92
Control _ Head kidney _2	5561239 (14.17%)	30303899 (77.21%)	97.99
Control _ Head kidney _3	5823611 (12.66%)	36266811 (78.81%)	98.2
3 dpi_ Head kidney _1	5995555 (14.67%)	31412470 (76.87%)	97.79

3 dpi_ Head kidney _2	4668466 (14.02%)	26009476 (78.11%)	98.16
3 dpi_ Head kidney _3	5534685 (13.99%)	30610945 (77.4%)	97.93
10 dpi_ Head kidney _1	6204518 (15.73%)	28809529 (73.06%)	96.6
10 dpi_ Head kidney _2	4851800 (13.56%)	27816099 (77.73%)	97.66
10 dpi_ Head kidney _3	4818814 (13.3%)	28270093 (78.02%)	98.01
Control_Spleen_1	4935796 (13.74%)	27895313 (77.64%)	97.95
Control_Spleen _2	4769616 (11.99%)	31464709 (79.13%)	98.33
Control_Spleen _3	5384352 (12.62%)	33497395 (78.51%)	98.1
3 dpi_ Spleen _1	5420901 (15.39%)	26700202 (75.8%)	97.82
3 dpi_ Spleen _2	5093933 (13.8%)	28863984 (78.17%)	98.12
3 dpi_ Spleen _3	5421149 (14.45%)	28750313 (76.62%)	97.88
10 dpi_ Spleen _1	5355795 (14.93%)	27159109 (75.71%)	97.73
10 dpi_ Spleen _2	5198840 (14.71%)	26876759 (76.07%)	97.81
10 dpi_ Spleen _3	5145686 (15.11%)	25916830 (76.1%)	97.83
Control_Liver_1	4775243 (12.14%)	31337959 (79.64%)	98.18
Control_Liver _2	5060475 (12.24%)	33095542 (80.03%)	98.29
Control_Liver _3	3988922 (10.35%)	31537686 (81.8%)	98.49
3 dpi_ Liver _1	3499716 (10.92%)	25588309 (79.87%)	97.92
3 dpi_ Liver _2	4756240 (10.41%)	37275425 (81.56%)	98.44
3 dpi_ Liver _3	5063196 (10.72%)	38469695 (81.42%)	98.67
10 dpi_ Liver _1	3473492 (10.91%)	25995568 (81.64%)	98.55
10 dpi_ Liver _2	4197048 (10.44%)	33072408 (82.25%)	98.52
10 dpi_ Liver _3	3454200 (9.93%)	28709153 (82.54%)	98.5
